# All-Weather 3D Self-Folding Fabric for Adaptive Personal Thermoregulation

**DOI:** 10.1007/s40820-025-01812-2

**Published:** 2025-06-12

**Authors:** Xiaohui Zhang, Yuheng Gu, Xujiang Chao, Zhaokun Wang, Shitong Wu, Jinhao Xu, Ziqi Li, Mengjiao Pan, Dahua Shou

**Affiliations:** 1 Future Intelligent Wear Centre, School of Fashion and Textiles, The Hong Kong Polytechnic University, Kowloon, 999077 Hong Kong People’s Republic of China; 2 Research Centre of Textiles for Future Fashion, The Hong Kong Polytechnic University, Hung Hom, Kowloon, 999077 Hong Kong People’s Republic of China; 3 Research Institute for Intelligent Wearable Systems, The Hong Kong Polytechnic University, Hung Hom, Kowloon, 999077 Hong Kong People’s Republic of China; 4 PolyU-Xingguo Technology and Innovation Research Institute, The Hong Kong Polytechnic University, Hung Hom, Kowloon, 999077 Hong Kong People’s Republic of China; 5 School of Mechanical Engineering, Northwestern Polytechnical University, Xi’an, 710072 People’s Republic of China

**Keywords:** Dual-mode fabric, 3D knitting, Personal thermal management, Thermal insulation, Radiative cooling

## Abstract

In the era of global climate change, personal thermoregulation has become critical to addressing the growing demands for thermoadaptability, comfort, health, and work efficiency in dynamic environments. Here, we introduce an innovative three-dimensional (3D) self-folding knitted fabric that achieves dual thermal regulation modes through architectural reconfiguration. In the warming mode, the fabric maintains its natural 3D structure, trapping still air with extremely low thermal conductivity to provide high thermal resistance (0.06 m^2^ K W^−1^), effectively minimizing heat loss. In the cooling mode, the fabric transitions to a 2D flat state via stretching, with titanium dioxide (TiO_2_) and polydimethylsiloxane (PDMS) coatings that enhance solar reflectivity (89.5%) and infrared emissivity (93.5%), achieving a cooling effect of 4.3 °C under sunlight. The fabric demonstrates exceptional durability and washability, enduring over 1000 folding cycles, and is manufactured using scalable and cost-effective knitting techniques. Beyond thermoregulation, it exhibits excellent breathability, sweat management, and flexibility, ensuring wear comfort and tactile feel under diverse conditions. This study presents an innovative solution for next-generation adaptive textiles, addressing the limitations of static thermal fabrics and advancing personal thermal management with wide applications for wearable technology, extreme environments, and sustainable fashion.

**Supplementary Information:**

The online version contains supplementary material available at 10.1007/s40820-025-01812-2.

## Highlights

- An innovative 3D self-folding fabric was fabricated by knitting technology to achieve dual thermoregulation modes through architectural reconfiguration between 3D and 2D states.
- In the warming mode, the fabric retains its natural 3D structure, providing high thermal resistance (0.06 m^2^ K W⁻^1^) by trapping still air. In the cooling mode, it transitions to a 2D flat state with coatings of thermal radiative management materials, achieving a cooling effect of 4.3 °C under sunlight by enhancing solar reflectivity and infrared emissivity, while reducing thermal resistance.
- The fabric demonstrates exceptional durability and washability, enduring over 1000 folding cycles, and is manufactured using scalable and cost-effective knitting techniques.

**Supplementary Information:**

The online version contains supplementary material available at 10.1007/s40820-025-01812-2.

## Graphical abstract

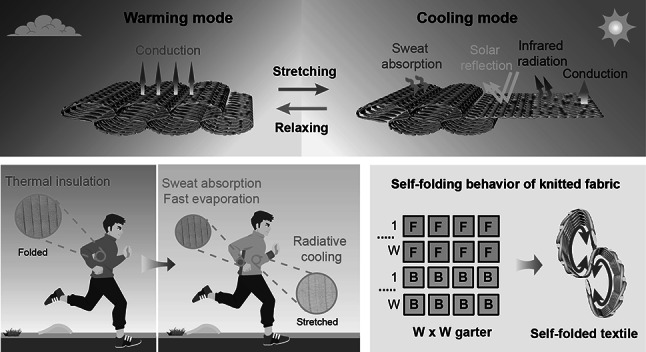

## Introduction

In recent years, managing personal thermal comfort has become a critical area of research, particularly in the context of global climate change and the increasing frequency of extreme weather events [1, 2]. The regulation of thermal conduction and convection in textiles has been widely explored as a means to achieve personal thermal comfort, while controlling radiative heat transfer between the human body and its surroundings has also emerged as an efficient approach [3–5]. However, most existing methods focus on regulating a single thermal transport mechanism with fixed thermal properties, limiting their adaptability to rapidly changing environmental conditions. In outdoor sports such as marathons or high-altitude activities [6], sudden extreme weather events can lead to rapid temperature fluctuations, posing significant risks to participants. These scenarios highlight the dual challenges faced by individuals in such environments: the need for effective solar protection during warm conditions and the risk of rapid heat loss during unexpected cold spells. Such situations underscore the limitations of traditional fabrics and clothing, which often lack the adaptability required to respond to dynamic and unpredictable environmental changes. This inability to adjust to rapid shifts in temperature can result in severe health risks, emphasizing the need for advanced textile solutions that can provide tunable thermal regulation to ensure safety and comfort in diverse conditions [7, 8]. Current solutions, such as adding or removing layers of clothing, are often impractical in outdoor or high-altitude settings, where carrying extra clothing is not feasible. Therefore, there is an urgent need to develop advanced fabrics and clothing systems that can dynamically adapt to complex and rapidly changing environments, integrating flexibility, and dual-mode thermal regulations to provide effective protection against both overheating and excessive cooling.

Motivated by this demand, much research has been conducted to design materials with precisely tunable thermal properties [9]. Materials with more than one layer often exhibit different thermal properties, which have dual mode. The desirable thermal performance can be achieved based on the high-selectivity spectral characteristics of each side by flipping the materials [7, 8, 10–15], transverse the direction [12] or automatically controlling the covering relations of layers [16]. Besides those dual mode textiles, there are some structures with dynamic thermal properties [17]. Inspired by the dynamic color-changing skin of coleoid cephalopods (squid, octopuses, and cuttlefish), researchers have designed materials with dynamic thermoregulatory, which can adjust their thermal emissivity [18–21]. Compared with conventional active cooling techniques, passive radiative management exhibits lower consumption of energy, lower emission of greenhouse gas and superior space conditioning efficiency [22–26]. However, those designs still have some limitations, including narrow modulation range, unstable structure, complex and costly methods and poor breathability. On the other hand, the aforenoted structures focused on the management of thermal radiative performance, not concerning the other heat transfer modes. Same of them may be used for building thermal management [27], which is not suitable for personal thermal management.

Knitting, as a versatile textile fabrication method, shares similarities with additive manufacturing techniques by transforming yarn, a 1D material, into intricate 2D or 3D fabric structures [28, 29]. By intertwining yarn through a series of loops and stitches [30], knitting produces fabrics that are not only flexible, durable, and intricate but also remarkably skin friendly [31]. The exceptional artistry of knitting lies in its ability to create complex patterns, textures, and structures, thereby offering infinite possibilities for design innovation and customization. The flexibility and adaptability of knitted fabrics make them ideal for a wide range of applications, from fashion and apparel to technical textiles [32–34], especially textiles for adaptive personal thermal management. Researchers have developed fabrics with adaptive thermal properties by modifying pore sizes through the deformation of moisture-responsive yarns or by utilizing dual-mode, flippable fabrics, thereby achieving dynamic personal thermoregulation [35–37]. However, these designs are traditionally based on 2D knitted fabrics, which deform primarily within their plane. Knitted fabrics can conform to the body's contours [38], providing comfort, freedom of movement, and breathability. Their inherent stretch and recovery properties make them suitable for various body types and activities. Notably, 3D knitting technology offers a remarkable set of capabilities that differentiate it from traditional knitting methods. This advanced technique excels in creating shapes that exhibit tunable compliance along engineered directions, allowing for spatially designed stiffness at high resolutions, down to the individual stitch level. 3D knitting can also utilize geometrically compliant interloping or interlaced stitch architectures to produce a wide range of flexible and stretchable bulk materials, even when the constituent yarns are inextensible [39].

In hot days and low latitude areas, intense solar radiation can increase the environmental temperature in a very short period of time. Hence, reflecting solar light to achieve efficient radiative cooling is important for people to stay in such conditions. While on cold days and high latitude areas, body warming is extremely important. Isolating thermal conduction can be an effective approach. If these two aspects can be combined, fabric and clothing with dual mode to achieve radiative cooling under intense solar light and less heat loss in low-temperature conditions can be created, which can help human adapt to a wide range of temperatures. Herein, we propose a feasible method to fabricate fabric with dual mode. The innovative concept is realized by preparing a self-folding 3D knitted fabric, which can transfer between 3 and 2D state. The unique 3D knitted structure of the fabric is designed to trap still air within its intricate folding structure, enabling highly effective thermal insulation properties. To enhance its versatility, specific portions of the fabric are coated with TiO_2_ and PDMS, which can be stretched to 2D state. This transformation enables the fabric to reflect solar light, emit infrared light, and improve heat conduction efficiency. In addition to its thermal features, the fabric exhibits a higher water vapor transmission ratio, facilitating enhanced moisture management. This characteristic allows for increased breathability and moisture to escape from the skin side, ensuring a dry and comfortable wearing experience. In summary, dual-mode fabric represents a versatile solution for coping with complex conditions, referring to thermal insulation, self-folding capability, reversible transformation and radiative management. By simply stretching the fabric system, the fabric can be effortlessly switched between the 3D and 2D states, adapting to different environmental requirements. This innovative design not only offers advanced thermal insulation and heat regulation but also prioritizes comfort, breathability, and adaptability, making it an efficient solution for modern textile applications.

## Experimental Section

### Materials

TiO_2_ particles and PDMS were bought from Sigma. The cotton yarn was purchased from Dongguan Hongxing Textile Trading Co., Ltd, whose specification is 20 S. The specification of the Coolmax yarn is 200 D. The tetrahydrofuran (THF) was purchased from Oriental Chemicals and Lab Supplies Ltd.

### Preparation of 3D Knitted Fabric

The 3D knitted fabric was fabricated by the flat weft knitting machine (STOLL CMS 822, E7.2) without any post-treatment to achieve self-folding behavior. The materials, including cotton and Coolmax yarn, are commercially available. This choice of yarn and knitting technology enables the large-scale production of 3D fabric at a cost comparable to other commercial garments. After fabrication, the fabric was washed to obtain a stable state as the cotton fabric is prone to shrinking due to their structure and composition. Washing cold water and low heat air-drying can stabilize the shape in further processes. To prevent shrinkage of fabrics after washing, all fabric samples underwent the aforementioned process after knitting to obtain a stable and consistent density. This stabilizes the fabric structure and prevents variations in fabric density that could arise from shrinkage in subsequent processes. The final density of the fabric is 5.7 wales cm^−1^ and 11.1 courses cm^−1^. And the fabric was also cleaned by ultrasonic to reduce residual impurities. The elongation ratio ($ER$) of the fabric was calculated by the following formula:

1 $${\text{ER}}\left( \% \right) = \frac{{\left( {L_{{\text{s}}} - L_{{\text{f}}} } \right)}}{{L_{{\text{f}}} }}$$

where ${L}_{\text{s}}$ is the length under fully stretched state, ${L}_{\text{f}}$ is the length under fully stretched state.

### Preparation of THF/PDMS/TiO_2_ Solution

Firstly, nanoparticles of TiO_2_ were dispersed into 20 mL of THF by ultrasonic treatment with 120 W for 30 min and magnetically stirred for another 30 min at room temperature. Then, 4 g PDMS A was added and magnetically stirred to obtain a homogenous suspension. After that, PDMS B was dissolved into 20 mL THF and magnetically stirred for 30 min. At last, the solution obtained in step 3 was added into that obtained from step 2 and magnetically stirred 12 h. The coating solution was completed. THF is used as a solvent to ensure the uniform distribution of nanoparticles in PDMS, as THF is compatible with PDMS and can effectively solvate the polymer chains. This compatibility helps in achieving a homogeneous mixture when nanoparticles are applied. The uniform distribution of TiO_2_ in the solution significantly impacts the coating uniformity. The weight ratio ($\text{WR}$) of particles was calculated by the following formula:

2 $${\text{WR}}\left( \% \right) = \frac{{W_{{\text{P}}} }}{{\left( {W_{{\text{P}}} + W_{{{\text{PDMS}}}} + W_{{{\text{THF}}}} } \right)}}$$

where ${W}_{\text{P}}$ is the weight of the particles, ${W}_{\text{PDMS}}$ is the weight of PDMS and ${W}_{\text{THF}}$ is the weight of THF.

### Preparation of the Dual Mode Fabric

The cleaned fabric was partly immersed into the coating solution, which was coated multiple times to produce a variety of samples with varying levels of nanoparticle content. After the coating process, all samples were placed on a plate at room temperature in a fume hood, waiting for the evaporation of the THF. Then, the samples were transferred to an oven for a curing process, which involved exposing them to temperatures of 80 °C for a duration of 4 h. The purpose of this curing process was to stabilize the coating and prevent the samples from breaking apart under normal wear and tears.

### Materials Characterizations and Measurements

The thermal resistance was measured by the sweating hotplate (Wenzhou Darong sweating hotplate (YG(B)) 606G thermal and moisture resistance tester). During the testing of the thermal resistance, the temperature of the hotplate and the chamber were set as 35 and 20 °C, respectively. The wind speed is 1 m s^−1^, and the humidity is 65%. During the testing of the moisture resistance, the temperature of the hotplate and the chamber were both set as 35 °C, respectively. The wind speed is 1 m s^−1^, and the humidity is 40%. The thermal images were taken by an infrared camera (Fluke Ti400). The temperature was collected by the handheld multi-channel temperature meter. Temperature data logging meter with USB Interface (Anbai, AT4208). The MIR emissivity was calculated according to MIR reflectance and transmissivity, measured by the FTIR instrument (PerkinElmer Spectrum 100). UV–vis–NIR reflectivity was measured by the UV–vis–NIR spectrometer (LAMBDA 1050, PerkinElmer). SEM images were taken by Tescan MIRA.

The thermal insulation properties of the dual mode applied for warming were conducted by putting the samples on a hotplate with adjustable surface temperature. A 5 × 5 × 2 cm^3^ cube cavity was dug out on a 20 × 20 × 10 cm^3^ foam. A 5 × 5 cm^2^ sample was inserted in this cavity with a thermal couple set on the inner surface of the sample. The fabric was fully stretched and sewn with foam to maintain the stretched state. The full foam with covered by aluminum foil, except for the cavity part, and PE film was used to cover the foam with Al foil to reduce influence from convection. The solar irradiance was recorded using a solar irradiance meter (TES132 Solar Power Meter). All the outdoor tests were conducted at the campus of Hong Kong Polytechnic University, Hong Kong.

The water absorption rate (%) and the evaporation rate (g h^−1^) were measured according to the Standards: GB/T 21655.1-2008 (Textiles—Evaluation of absorption and quick-drying). Water absorption rate ($A$) indicates that the percentage of water absorbed by the sample to the original mass of the sample when it is completely immersed in water and taken out without dripping. The evaporation rate represents that a certain amount of water is dropped on the sample and hung in a standard atmosphere for natural evaporation. The evaporation mass per unit time on the time evaporation curve is within a linear interval.

3 $$A\left( \% \right) = \frac{{\left( {m - m_{0} } \right)}}{{m_{0} }}$$

$m$ is the weight of the sample after soaking and dripping water. ${m}_{0}$ is the initial dry weight of the sample.

The washing tests were conducted under the modified standard: ISO 6330:2021, with a washing temperature of 20 °C and 5 washing cycles. The weight variations before washing and after 5 washing cycles were recorded. The air permeability was measured by SDL international Textile Testing Solutions. The tensile strength test was conducted under the D5035 Textile Breaking Strength/Elongation Strip Method by Instron 4411 using a universal testing (Instron-4411, INSTRON CORPORATION Co., LTD). The rectangle sample fabric with size of 7.5 cm × 5 cm were stretched at a constant rate of 300 mm min^−1^. Each sample was measured repeatedly five times. According to the ASTM E96 with modification, the water vapor transmission was calculated based on the water loss from a glass bottle filled with deionized water in the condition with a humidity of 40% and a temperature of 26 °C. The water vapor ratio ($WVR$) can be calculated by the following formula:

4 $${\text{WVR}}\left( \% \right) = \frac{{\left( {W_{0} - W_{i} } \right)}}{{\left( {W_{0} - W_{g} } \right)}}$$

where ${W}_{0}$ is the total weight of the bottle, and water is added. ${W}_{i}$ is the total weight of the bottle and the water after testing. ${W}_{g}$ is the weight of the bottle.

## Results and Discussion

### Concept of the Dual Mode Knitted Fabric

**Fig. 1:**
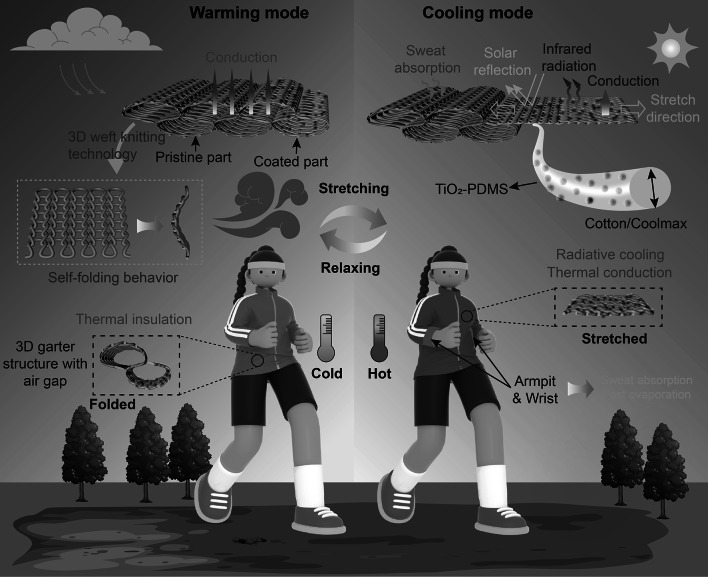
Schematic of dual mode textile demonstrating cooling and warming mode. In warming mode, fabric exhibits a relaxed 3D structure for enhanced thermal management. Increased fabric thickness creates numerous air pockets that act as barriers to heat flow, reducing heat loss from skin. The ideal for cold environments, fabric retains body heat and keeps wearer warm and comfortable. 3D structure adds to user comfort. In cooling mode, fabric is fully stretched, covering skin surface efficiently. This action increases contact surface area and enhances fabric's reflection of solar radiation, reducing heat absorption. Fabric also emits infrared radiation to dissipate excess body heat, promoting cooling. Additionally, enhanced heat conduction capabilities aid in heat transfer away from body. This dual functionality provides comfort and temperature regulation across varying environmental conditions

As illustrated in Fig. 1, the dual mode knitted fabric with self-folding behavior is designed to enable adjustable radiative cooling and warming, thus effectively adapting to various environmental conditions. This innovative fabric operates by dynamically transforming between its 3D and 2D states, providing a versatile solution for temperature regulation. During warming mode, the fabric maintains its original 3D self-folding structure as a relaxed state, which implies that there is no external force acting on the fabric. The unique 3D structure of the fabric results in a thick fabric with trapped still air within its intricate weave cavities. This trapped still air plays a crucial role in the fabric’s thermal management capabilities. Air, being a poor conductor of heat compared to fibrous polymers and composites, provides an effective insulating layer [1]. The low thermal conductivity of the stationary air reduces heat transfer from the skin to the environment, thus conserving body warmth. The design of the fabric effectively minimizes thermal convection by restricting airflow between the separate air-filled cavities. In the folded state, the limited air movement within these cavities reduces convective heat transfer, contributing to higher thermal resistance. This property is particularly advantageous in cold climates, as the fabric minimizes heat loss, thereby maintaining a comfortable warmth for the wearer. Additionally, the structural design of the fabric not only enhances thermal insulation but also contributes to its overall durability and structural integrity. Consequently, the fabric in its relaxed state exhibits substantial thermal insulation capabilities, reducing heat loss from the human body in cold conditions. In cooling mode, the coated part of the fabric is specifically engineered to optimize thermal management by leveraging advanced materials. This section of the fabric contains a coating of TiO_2_ and PDMS, which are manually stretched to cover the skin, transforming the fabric into its cooling configuration. TiO_2_ is selected for its well-documented near-infrared (NIR) reflective properties [40]. This characteristic is crucial because NIR reflection significantly reduces solar heat absorption, preventing excessive internal energy buildup from solar radiation. By reflecting NIR radiation, the fabric lowers the amount of heat entering the system, maintaining a cooler microclimate around the body. Furthermore, the coatings with PDMS are designed with high emissivity, which enhances IR emission. This feature is complemented by the fabric's high mid-infrared emission (8–13 µm), which facilitates the transfer of heat through what is known as the “atmospheric window.” This window is a range of wavelengths where Earth’s atmosphere is particularly transparent to infrared radiation, allowing heat to efficiently radiate into outer space. By emitting heat through this window, the fabric effectively dissipates excess body heat, enhancing the cooling effect. Simultaneously, as the coated part is manually stretched, the fabric transitions from its original 3D (folded) state to a 2D (flat) state. This transformation not only maximizes the surface area in contact with the skin but also decreases the thickness of the fabric, thereby enhancing heat conduction away from the skin. The reduced thickness allows heat to be conducted more efficiently, further supporting the cooling mechanism. For the dual mode knitted fabric, the pristine part remains unchanged during the adjustment process. This stability is achieved by connecting the edges of the fabric using non-stretchable yarn, which preserves the integrity and shape of the fabric’s original form. The non-stretchable yarn acts as a structural anchor, preventing deformation and ensuring that the pristine part maintains its intended properties throughout the various transformations. The pristine section is strategically folded together in areas like the wrist and armpit. These regions are chosen for their high likelihood of moisture accumulation, as they are common areas for sweat production. By concentrating the pristine part in these locations, the fabric effectively absorbs sweat, enhancing comfort and usability. The material's ability to manage moisture is crucial in maintaining a dry and pleasant experience for the wearer, especially during physical activities or in warm environments. Additionally, the non-stretchable yarn not only contributes to maintaining the structure but also supports the fabric’s functional design by allowing other sections of the fabric to dynamically transform as needed. This innovative combination of structural stability and moisture absorption ensures that the fabric performs efficiently across its cooling and warming modes, addressing both thermal and comfort needs. To illustrate the practical application of the knitted structure, Fig. S2 presents a shirt designed and fabricated using the proposed fabric. In summary, in warming mode, the pristine part and the coated part of the dual-mode fabric collaborate to offer effective thermal conduction and weakened thermal convention for warmth. The fabric's folded length is designed to fully cover the skin and is secured with buttons or fasteners, maintaining its stretchability and ensuring a wear fit. This configuration enhances the insulation properties by trapping heat. Conversely, in hot and sunny conditions, the coated part is fully stretched, while the pristine part remains unaffected by any external force. This stretching allows the fabric to cover the skin completely, optimizing its cooling capabilities. The coating features enhance the fabric's ability to reflect sunlight and manage heat through radiative processes. Through its ability to transition flexibly between different states, this dual-mode fabric successfully achieves radiative cooling and warming, adapting efficiently to diverse environmental conditions.

### Fabrication of the Foldable Knitted Fabric

**Fig. 2:**
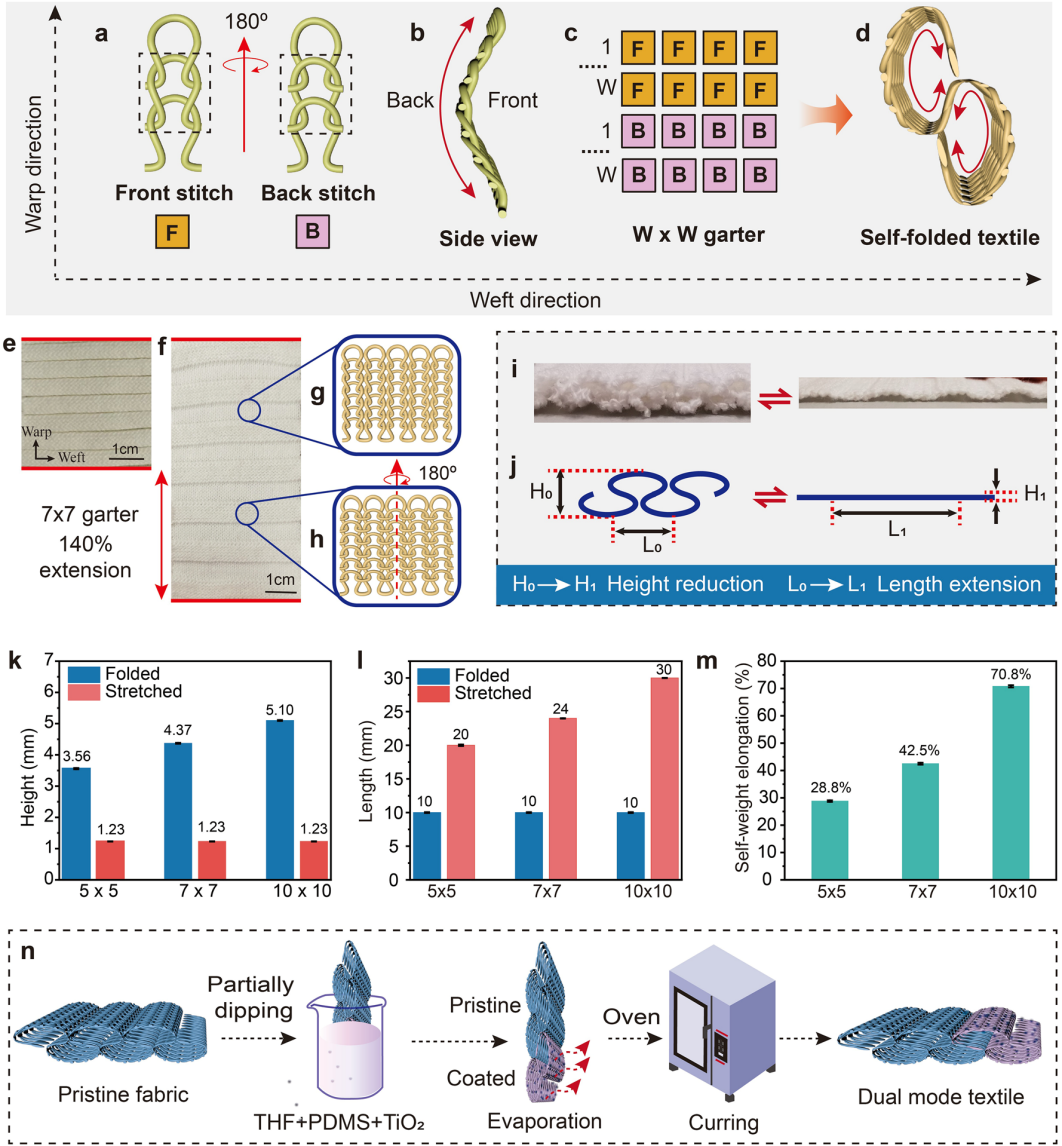
Design and fabrication of dual mode fabric. **a** Face view of single jersey knit. The front stitch, where the loop head is under the loop legs. The back stitch, where the loop head is on the loop legs. **b** Side view of single jersey knit. The whole structure will curl from the back side (back stitch side) to the face side (front stitch side). **c** Combination of front and back stitches. **d** Side view of the curled fabric with combination of front and back stitches. **e** Photography showing the surface of the folded pristine fabric. **f** Photography showing the surface of the fully stretched pristine fabric. **g, h** Schematic of the front and back stitches. **i, j** Photography and schematic showing the side view of the folded (0% strain) and stretched state (100% strain) of the 3D fabric. The deformation of the 3D fabric is accompanied by the height and length variations. **k** Height of the 3D structure of the fabric under 0% and 100% strain. **l** Length of the 3D structure of the fabric under 0% and 100% strain. **m** Self-weight elongation. Weight of 5 × 5, 7 × 7 and 10 × 10 garter under folded state is 919.15 g m^−2^, 1057.51 g m^−2^ and 1254.72 g m^−2^. **n** Coating process of the dual mode fabric

Knitting is a traditional textile fabrication method, which can turn yarn, a 1D material, into 2D and 3D fabric, which can also be viewed as an additive manufacturing technology [28]. The stitch-by-stitch programming method allows for more complicated structures that are durable, soft and smart. In order to design the dual mode textile, the material selection and fabrication approach of the fabric is important. The knitted fabric was fabricated by the weft knitting machine with two needle beds. The cotton yarn, known for its durability and stability, offers support for the folding behavior of the fabric. Additionally, the Coolmax yarn was incorporated for its moisture-wicking properties, enabling rapid perspiration and evaporation [41–43]. This strategic combination of materials ensures a balance between structural support and enhanced moisture management, making the fabric suitable for a range of environmental conditions and activities. For knitted fabric loops, the term “course” used in the context of weft-knitted fabric indicates a horizontal row of stitches along the weft direction, while the term “wale” refers to a vertical column of stitches along the warp direction. For the single jersey structure shown in Fig. 2a, the whole structure will curl from the back side to the face side as the yarn in the loops is bent. The side view of the single jersey structure, shown in Fig. 2b, curls from the front side to the back side. The curling mechanism of the knitted fabric is explained in Supplementary Materials. Figure S3a shows the front stitch in a fabric loop, where the loop legs (the grape part) are on the loop head. Figure S3b is the back stitch in a fabric loop, where the loop legs on the loop head (orange part). The torque of the bended yarn in the fabric always tries to recover to the relaxed state [44]. Thus, the head of the loop will curl to the face side, leading to the curling of the whole fabric to the face side. Figure S3c displays the single jersey with face side as the upper surface. In this configuration, the front stitch exhibits a natural forward roll of both the top and bottom edges, resulting in a concave shape. Figure S3d displays the single jersey with back side as the upper surface. Here, the top and bottom edges roll naturally backwards, creating a protruding appearance. By integrating these opposing curvatures of the stitches, a self-folded structure of the entire fabric can be achieved. Actually, front and back stitches are structurally symmetric. The side from which they are viewed determines their nomenclature in the knit structure.

To knit the proposed 3D knitted fabric, the front and back stitches are combined in one cohesive unit, as illustrated in Fig. 2c. If the course counts of the front and back stitches are the same, which is “W”, the fabric can be called as *W* × *W* garter. Figure 2d presents the side view of the curled structure, offering a perspective on the dimensional characteristics and textural properties of the self-folded fabric. This integration of front and back stitches enables the creation of intricate patterns and structures that contribute to the fabric's unique properties and functionalities. The loop structure of one unit is illustrated in Fig. S3e. The corresponding knitting notation is provided in Fig. S3f. Each single unit comprises 14 courses, consisting of 7 front stitches and 7 back stitches, referred to as a 7 × 7 garter. The first “7” denotes the number of courses of the front stitches, while the second “7” indicates the courses with back stitches. Figure S4a displays the loop structures formed by front and back stitches, respectively. Front and back stitches are structurally symmetric. Figure S4b depicts the surface photography of the samples. This self-folded fabric can be created through the combination of front and back stitches, known as garter stitch. The distinct edges can be observed between the front and back stitches, highlighting the unique characteristics of the garter stitch pattern.

This structure is knitted by a two-bed weft-knitting machine with a loop transferring between two beds. By utilizing this technique, self-folded fabrics can be manufactured that incorporate both front stitches and back stitches on the same continuous side of the fabric. Typically, the counts of the front and back stitch in one single unit are generally the same to maintain stability of the fabric. Ensuring symmetry in the number of courses knitted on different needle beds leads to a folded 3D fabric that is symmetrical as well. Simultaneously, symmetry contributes to a more stable and balanced fabric structure. Symmetry is the fundamental aspect of self-folded fabric design, and ensuring an equal distribution of stitches is integral to achieving the structural integrity and aesthetic appeal of the structure. Conversely, if they are not equal, the structure will be asymmetric, potentially resulting in unstable structure. In conclusion, the self-folding behavior of the weft-knitted fabric is structure-driven behavior, which can be created by the arrangements of front and back stitches during knitting process. The inherent self-folding behavior manifests when the fabric leaves the knitting machine, eliminating additional post-processing procedures required to achieve unique structure [45]. The self-folded knitted fabric has great potential in developing functional and innovative textiles, opening the doors to create dynamic, adaptable and versatile products. Weft knitting is a highly developed textile fabrication technique that enables the production of a wide variety of fabrics on a large scale. The garter structure offers the advantage of allowing the use of commercially available fibers and yarns. This simplicity in knitting structure not only facilitates ease of manufacturing process but also enables efficient large-scale production of textiles. Figure 2e presents the surface view of the 3D fabric in a 7 × 7 garter, where the horizontal lines are prominently visible. This pattern showcases the fabric's textured surface, contributing to its structural and aesthetic qualities. When the fabric is fully stretched along the warp direction, as depicted in Fig. 2f, the length increases, illustrating the fabric’s extensibility and flexibility. This transformation highlights the versatility of the fabric in adapting to different shapes. The detailed views in Fig. 2f also show the edges between the front (Fig. 2g) and back stitches (Fig. 2h), providing a clear visual distinction and demonstrating the intricate construction of the knitted fabric. These images collectively emphasize the dual-mode fabric's ability to maintain structural integrity while transitioning between its relaxed and stretched states.

Figure 2i, j presents the photography and diagram depicting the cross section of the folded structure at 0% strain and the fully stretched structure at 100% strain, with the strain applied along the warp direction. It appears that when the fabric is stretched, its length along the face side and its height along the thickness direction both undergo variations. This transformation demonstrates the fabric's ability to adapt its structure in response to applied strain, optimizing its functionality for either warmth or cooling as needed. In this research, samples, 5 × 5 and 10 × 10 garter, were also fabricated. Notably, there is a height difference between samples, resulting from the variations of courses in one unit. The surface of those fabrics is also different. There are width disparities between samples due to the diverse courses. Specifically, fabrics with more courses tend to exhibit larger widths on the surface and higher vertical height. The length and height of the fabric under folded state and fully stretched state can be observed in Fig. 2k, l. When comparing the length of fabric samples with the same initial length in the folded state, 10 × 10 garter exhibits the greatest increase in length when stretched, followed by the 7 × 7 garter and 5 × 5 garter. Assuming an original length of 10 mm in the folded state, the length of the 5 × 5 garter, 7 × 7 garter, and 10 × 10 garter after stretching are 20, 24, and 30 mm, respectively. This indicates that the higher the garter count, the greater the elongation capability of the fabric when subjected to stretching, highlighting the influence of the garter pattern on the fabric's dimensional changes and properties. The elongation ratio can be 100%, 140%, and 200%, calculated by the formula demonstrated in the Experimental sections. It is observed that as the number of courses in a single unit increase, the length of the fabric after stretching also increases. Similarly, 10 × 10 garter also has the greatest height/ thickness in the folded state. Specifically, the height of 5 × 5, 7 × 7 and garter in folded state are 3.9, 5.1, and 6.3 mm, respectively. After stretching, all the height of the fabric is 1.8 mm as the height, aligning with the thickness of the 2D fabric. Furthermore, the height of the fabric correlates with the number of courses in a single unit, showing that an increase in the garter count leads to a higher fabric height. However, it does not mean that the fabric with more units exhibits better performance. On the one hand, if there are too many courses, the force generated by the different loop directions can be weakened, as the strongest force typically occurs at the edges of the fabric. The middle part may lack adequate support, causing the fabric to become loose or lose its original shape, and the fabric will not be folded. On the other hand, the elongation of the fabric increases with the increased numbers of courses in one unit. However, the larger elongation of the fabric does not mean better performance since the fabric has inherent weight. It is inevitable that when excessive courses are included within a unit, a downward force will generate, and then stretching the fabric, which is not desirable. In order to test the self-weight elongation of fabrics due to their own weight, experiments were designed, showing in Fig. S6. The testing results are shown in Fig. 2m. When the length of the samples is the same, 10 × 10 garter has the largest length change rate under the own weight, followed by 7 × 7 garter and 5 × 5 garter. The possible reason is that more courses in one unit can lead to loosening force between loops, which are easy to stretch due to their own weight. Thus, the length change rate of the fabrics under their own weight should be considered as the loose structure of the fabric may not only influence the thermal properties but also the aesthetics of garment. When designing and manufacturing fabrics, it is essential to consider the number of courses, loop directions, and overall structure comprehensively to achieve optimal performance. Additionally, the results suggest that when the fabric is oriented with the "wave" pattern horizontally, it may expand under its own weight. To prevent this and ensure stability, the fabric should be positioned vertically, as illustrated in Fig. S2. Figure 2n illustrates the coating process of the 3D fabric. The folded fabric is partially coated by immersing part of the fabric into the PDMS, TiO_2_and THF mixed solution. After evaporation of the THF and curing of PDMS, the dual mode fabric is well prepared. The details of the preparation of the THF/PDMS/TiO_2_ solution can be found in the Experimental Sections below. As the dual mode fabric is partially coated, the ratio of pristine and coated part should be calculated. Figure S5 presents a simplified mathematical model that visualizes the deformation process of the dual-mode fabric. The dual-mode fabric, being partially coated, requires a careful balance in the ratio between the coated and pristine sections to achieve optimal performance. It is crucial that the coated part of the fabric is sufficiently long to cover the skin when stretched, while also avoiding excessive coating that could lead to unnecessary weight increase of the overall fabric. In order to determine the ideal ratio of pristine to coated sections in the dual-mode fabric (specifically a 7 × 7 garter), precise calculations have been conducted. According to the results, the optimal ratio for maximizing performance and comfort is determined to be 7:5, ensuring a balance between coverage, weight distribution, and functionality. This calculated ratio not only enhances the fabric's ability to transition between its 3D and 2D states effectively but also ensures that the coated areas provide the desired functionalities without compromising the overall comfort and wearability of the fabric.

### Thermal Performance Measurement of the Dual Mode Knitted Fabric

**Fig. 3:**
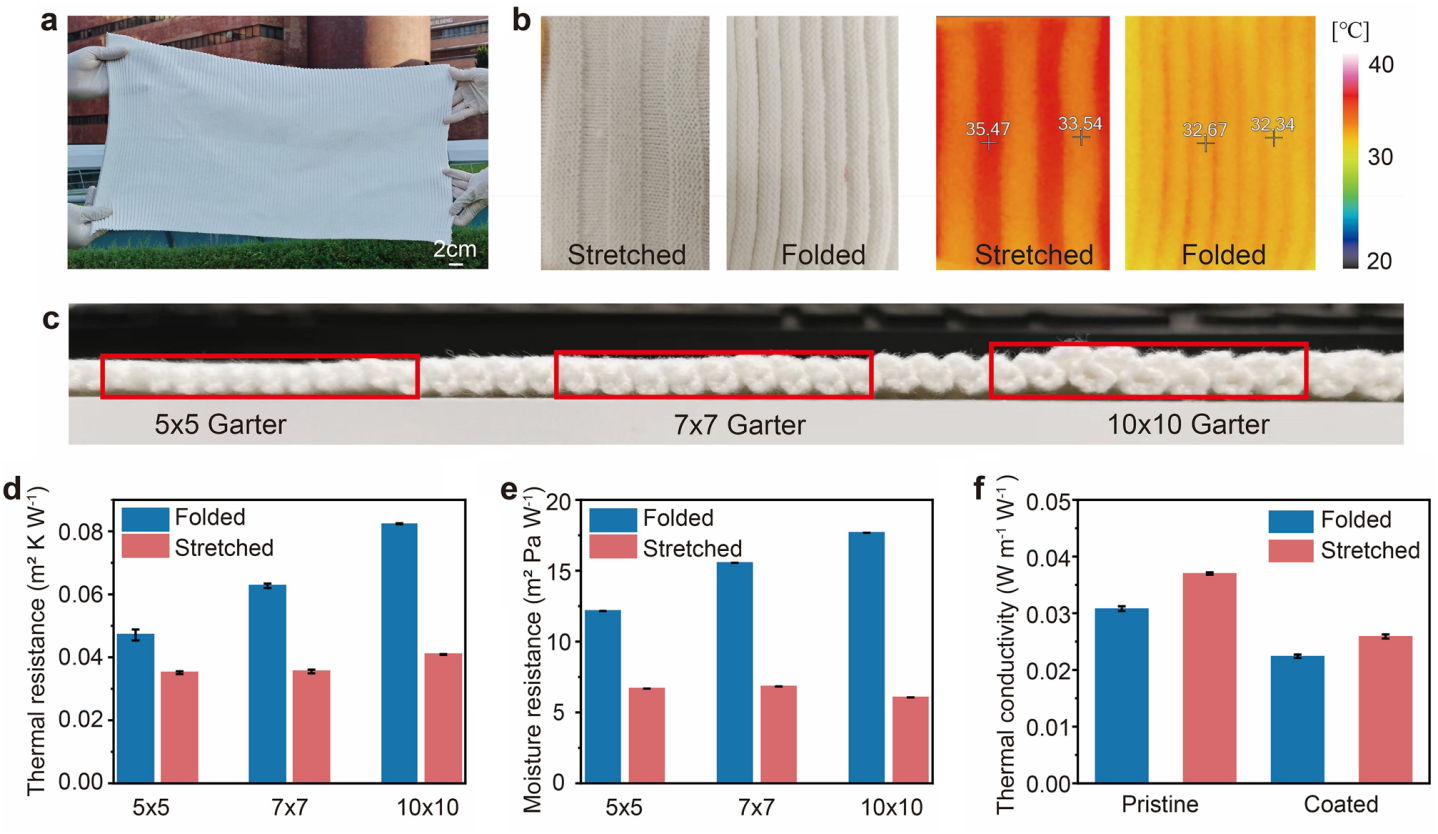
Thermal properties of the fabric in warming mode. **a** Photography of the textile for warming mode, showing the dual mode fabric by scalable knitting method. **b** Photography and thermal images of fabric under stretched and folded state when set on hotplate. **c** Photography of fabric with 5 × 5, 7 × 7 and 10 × 10 garter. **d** Thermal resistance of the pristine fabric in folded state and fully stretched state, including 5 × 5, 7 × 7 and 10 × 10 garter. **e** Moisture resistance of the pristine fabric in folded state and fully stretched state, including 5 × 5, 7 × 7 and 10 × 10 garter. **f** Thermal conductivity of the pristine and coated fabric (7 × 7 garter) in folded and fully stretched state

In order to evaluate the working efficiency of the dual mode fabric, some experiments are designed to test the thermal performance under warming and cooling mode. The proposed fabric whose size is 0.52 m × 0.24 m is displayed in Fig. 3a, showcasing its scalable production, which is also the photography of the proposed fabric for warming mode. Figure 3b shows the optical and thermal images of the pristine folded fabric (right) and pristine stretched fabric (left) on the hotplate. The temperature difference can reach more than 1 °C between the stretched and folded fabric, ensuring the warming property of the 3D fabric. Figure 3c displays the cross section of three samples, 5 × 5 garter, 7 × 7 garter and 10 × 10 garter. It is clear that there is a thickness variation between them. The thermal and moisture resistance is shown in Fig. 3d, e. The testing setup can be found in Fig. S7. For 7 × 7 garter, when the folded fabric is stretched, the thickness can reduce from 4.37 to 1.23 mm, followed by the decrease of thermal resistance from 0.0627 to 0.0355 m^2^ K W^−1^. With the increase of the total courses in one single unit, the thermal and moisture resistance of the folded fabric increases. However, the thermal and moisture resistance is similar when the fabric is fully stretched, proving that the fully stretched fabric is a thin layer, and the height is equal to the thickness of single course. As the count within a single unit increase, the cavities between the self-folding fabric layers also expand. Our proposed textile leverages a 3D self-folding structure that adapts for thermal management. In the folded state, depicted on the left side of Fig. 2j, the fabric exhibits a wavelike structure. The cavities formed between these waves are filled with stilled air, which significantly enhances thermal insulation of the entire fabric system. This design effectively minimizes thermal convection by restricting airflow between the separated cavities. Conversely, when the fabric is stretched, the wavelike structure transitions into a flattened state with reduced thickness, thereby decreasing thermal resistance. To investigate the influence of the coating on thermal properties, the thermal conductivity of the pristine and coated fabric was measured with results shown in Fig. 3f. Regarding the comparison between pristine and coated fabrics in the folded state, our results indicate that the coated fabric demonstrates lower thermal conductivity than its pristine counterpart. This trend remains consistent even when the fabric is stretched. The reduction in thermal conductivity for the coated fabric can be attributed to the coating's ability to further restrict airflow and enhance insulation properties. Consequently, in both folded and stretched states, the coated fabric exhibits lower thermal conductivity compared to the pristine version. The dual mode fabric was also put on a hotplate with temperature 35 °C. Figure S8a, b illustrates the optical and thermal images of the dual mode fabric with the pristine part on the left side and the coated part on the right side. The thermal image of the dual mode fabric shows similar temperature, indicating that the coatings have quite a smaller influence on the thermal insulation performance under the warming mode, which can be ignored. The thermal images of the three samples under folded state were also collected when they are put on human arms, which can be found in Fig. S8c. The surface temperature difference is evident, where 5 × 5 garter has the highest surface temperature and 10 × 10 has the lowest one.

**Fig. 4:**
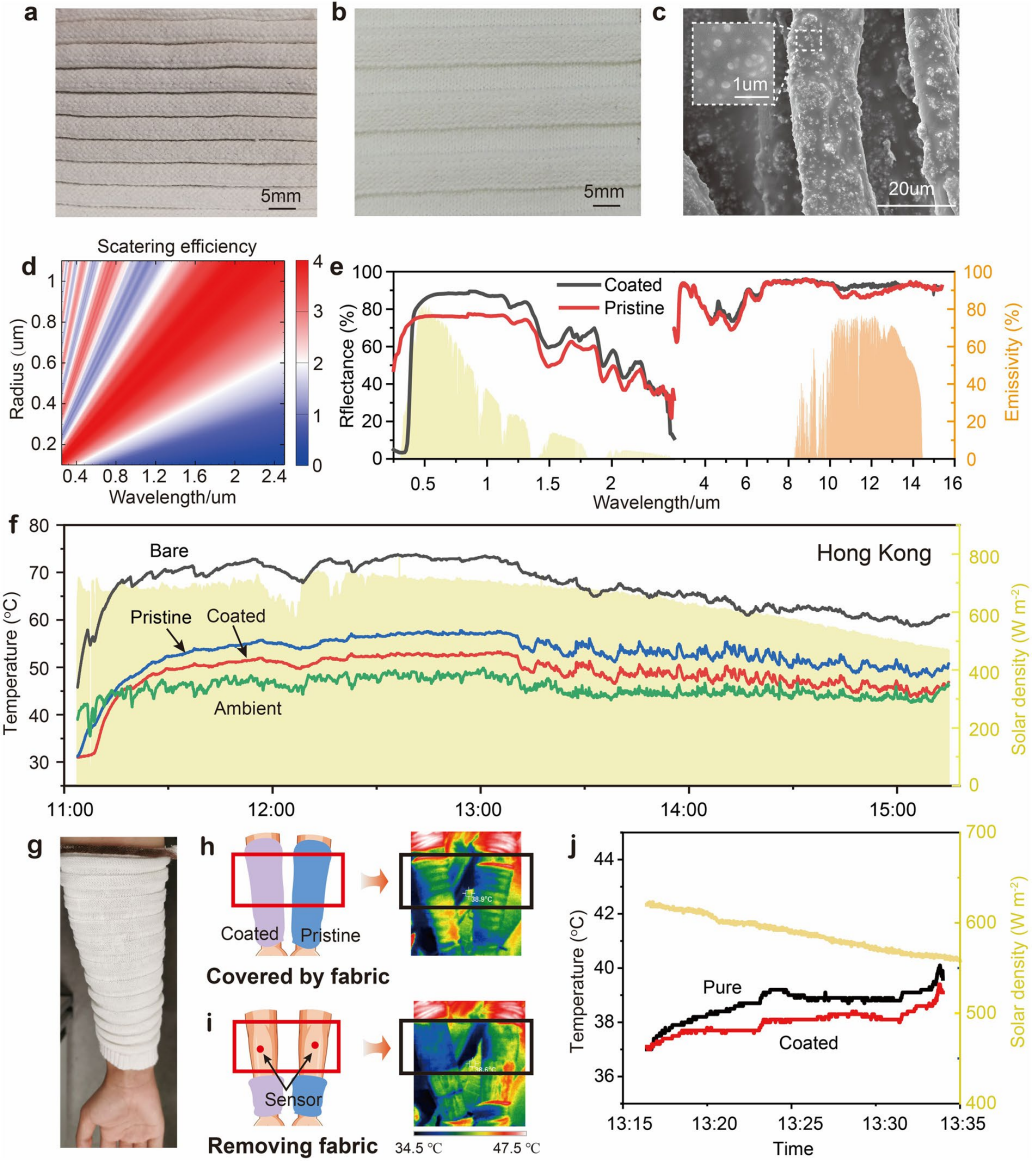
Optical and thermal properties of the fabric in radiative cooling mode. **a** Photography of the coated folded fabric. **b** Photography of the coated stretched fabric. **c** SEM image of the coated fabric. **d** Scattering efficiency of different particle radius under 0.25–2.5 µm. **e** Visible and near-IR reflectance spectra of the fabric in fully stretched state, including coated fabric for radiative cooling and pristine fabric as the control sample. Mid-IR reflectance and emissivity spectra of the fabric in fully stretched state, including coated fabric for radiative cooling and pristine fabric as the control sample. **f** Solar density from 11:30–15:50 in Hong Kong, 28th, 9, 2023. And the temperature measurement of the radiative cooling performance of the fully stretched coated fabric, fully stretched pristine fabric and the ambient temperature. **g** Human arms with sleeves made by coated fabric. **h** Schematic and thermal image of arm covered by coated (left) and pristine (right) fabric. **i** Schematic and thermal image of arm collected immediately after removing fabric under solar exposure. **j** Skin temperature collected during human arm covered by fabric exposed to solar light

**Fig. 5:**
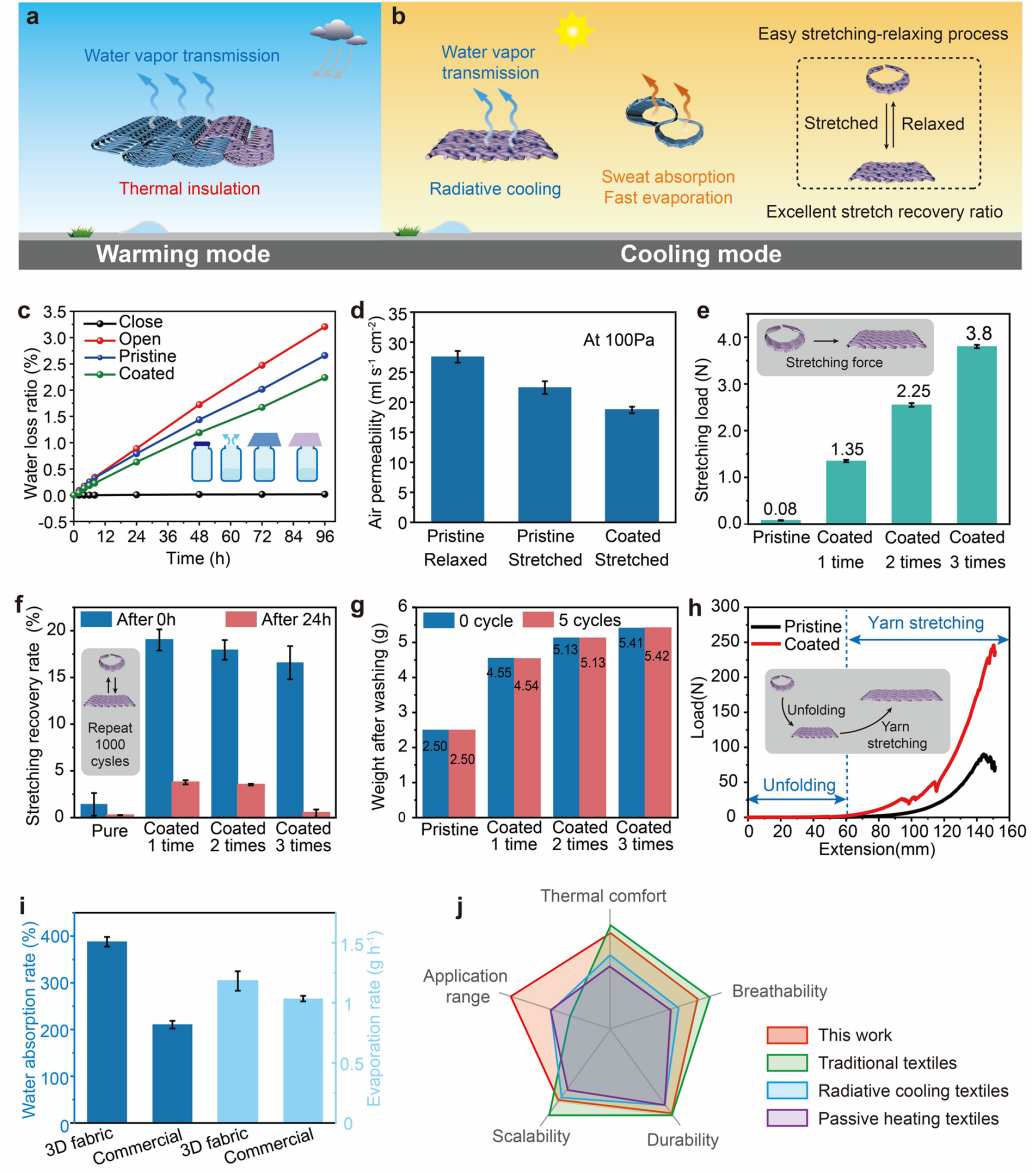
Wearability of the proposed fabric. **a, b** Schematic of the clothing made by dual mode fabric. **c** Water absorption and evaporation rate of the dual mode fabric and commercial fabric. **d** Air permeability of pristine folded fabric, pristine stretched fabric and coated stretched fabric (4.5%, 2 times). **e** Stretching load required to expand 2.5 cm of the fabric along the warp direction. **f** Stretching recovery rate of the fabrics after stretching 100 cycles after 0 h and 24 h. **g** Weight of fabrics after washing. **h** Tensile strength of the fabric (4.5%, 2 times). **i** Comparison of water absorption rate and evaporation rate of the 3D fabric (4.5%, 2 times) and commercial fabric**. j** Radar chart for comparison among various textiles

Figure 4a, b shows the coated folded and stretched fabric. Under radiative cooling mode, the coated part of the fabric is fully stretched to cover skin, aiming to reflect the solar light. Simultaneously, the fully stretched fabric has quite a smaller thickness, which can enhance heat transfer to the external environment. And the SEM of the coated yarn can be found in Fig. 4c. Figure S9 demonstrates SEM images of fabric with different particle weight ratio and coating times. It is evident that the surface of the pristine fiber is smooth, while the surface of the coated fiber (4.5%, 2×) has adhered to the TiO_2_ particles and PDMS. However, the larger weight ratio of TiO_2_ may lead to aggregation, weakening the radiative performance. To avoid aggregation of particles, the method of multiple coating times to increase nanoparticle content has been adopted. In order to test the optimal weight ratio of the nanoparticles, the solutions with different contents of nanoparticles were fabricated. There are no particles attached to the pristine fiber. As the TiO_2_ weight ratio is 4.5% to 10%, coating 1 time, the content of particles attached to the fiber surface significantly increases. However, when the weight ratio reaches 10%, there is aggregation of particles. To prove that multiple coats can increase the number of particles, fiber with a weight ratio of 4.5% and coating 1 time was also collected. It is obvious that the particles on the surface of the fiber coated twice are significantly more than those coated only once. Furthermore, the particles on the surface of the fiber coated twice with a weight ratio of 2.5% were more than that coated only once with weight ratio of 4.5%. Thus, the weight ratio of particles, 4.5%, coating 2 times was adopted in this study. Actually, 3 times of coating were also implemented. However, due to the larger load required to stretch the folded fabric, they were not applied, which is explained in Fig. 5d.

The average values of reflectance in the range of 0.25–2.5 µm and the average values of emissivity in the range of 2.5–15 µm are illustrated in Fig. 4e. To ensure the uniformity of the coating, tests were conducted at different locations of the same sample as well as on different samples. The reflectance difference between the coated (89.5%) and the pristine fabric (78%) can reach 11.5%. And the emissivity of the coated fabric in the range of 8–13 µm also reach 93.5%. This relies on the high scattering abilities of TiO_2_ particles in the visible and near-infrared wavelengths. PDMS, as a transparent elastomer, maintains high light transmittance in both visible and infrared ranges. This property allows PDMS to provide good mechanical support without hindering the scattering and radiative functions of TiO_2_ particles. Actually, the reflectance of the fabric with different particle sizes and different coating times were both measured, shown in Table S1. The results indicated that the reflectance of the fabric with different particle sizes is similar without larger difference. The possible reason is that the proposed 3D knitted fabric has larger pore size between yarns even when they are coated, allowing more solar light to pass through, leading to smaller difference between samples. The fabric with 3 times of coating has a little higher reflectance than the one only coated 2 times, indicating the feasibility of the multi-coating method. However, more coating times also lead to stiffer fabric, which not only influences the wearability of the fabric but also the external load required to stretch the folded fabric. The load increases when the coating time increases. After comprehensive comparison of the results, coating 2 times is much more suitable than others, which has both higher reflectance and easier deformation process. The finite-difference time-domain (FDTD) simulation is applied to study the effects of particle size on the optical properties. The results of the scattering efficiency can be found in Fig. 4d.

After studying the optical properties of the dual mode fabric, the outdoor thermal measurement was conducted. Figure S10 shows the setup to evaluate outdoor and indoor thermal measurement. Figure 4f is the results of a 4 continuous hour test of the solar density and the temperature variations of the coated and pristine fabric. As the radiative cooling mode of the dual mode fabric works when the coated fabric is fully stretched. Thus, the testing of the radiative cooling performance was conducted with the fully stretched fabric. The temperature difference between the coated fabric and the pristine fabric can reach 4.3 °C. The radiative cooling performance of the proposed fabric under solar simulator was also conducted, whose results were shown in Fig. S10. The temperature difference between the coated fabric and the pristine fabric can reach 4.9 °C. TiO₂ particles are effective at scattering sunlight, thereby significantly reducing heat absorption on material surfaces. They also possess excellent emissive properties in the thermal infrared range. When combined with the high light transmittance of PDMS in both visible and infrared spectra, these coatings on the fabric surface enhance the material’s ability to efficiently release heat into outer space. This results in a lower temperature for objects covered by coated fabric. Lastly, the radiative cooling performance of the dual mode fabric was assessed in the wearable system to manage the temperature of human body. Sleeves were fabricated and fitted to the arm of a human subject, shown in Fig. 4g. The real images and schematic of the forearms covered with dual mode textile-based sleeves are shown in Fig. S11. And then, the temperature change of the arms covered by coated fabric and pristine fabric were recorded by the thermal images and the temperature values measured. Figure 4h shows the schematic and thermal image of the human arm covered by coated (left) and pristine (right) fabric. Figure 4i shows the schematic and thermal image of the human arm immediately after removing fabric under solar exposure. As shown in the black box, the bare skin temperature of the arm on the left side is smaller than the one on the right side. The effect of the radiative cooling can also be proved by the temperature difference shown in Fig. 4j. The maximum temperature difference of the skin reaches more than 1 °C.

Having confirmed the thermal properties of the dual mode textile, the wearability of the proposed fabric was also evaluated to ensure the wear comfort. Figure 5a, b shows the schematic clothing made by the dual mode fabric. Under warming state, the clothing is folded with larger thickness to reduce heat loss. While under cooling mode, the fabric is fully stretched with the existing folded part accumulates to the side and wrist of human, which can absorb the sweat. The results in Fig. 5c show that the water loss ratio of the coated fabric is smaller than that of the pristine fabric and the bottle without cover, while the difference is smaller when compared with the bottle with close end, which has almost zero water loss. The proposed fabric in radiative cooling mode still has excellent water vapor transmission, allowing more moisture to escape from skin to ensure thermal comfort. The setup for the test can be found in Fig. S12. The air permeability of fabrics was measured at 100 Pa. As shown in Fig. 5d, for pristine fabric, when the fabric transfers from folded state to the fully stretched state, the air permeability reduces from 27.56 to 22.44 mL s cm^−2^, almost 18.58%. The air permeability of the fully stretched coated fabric is 18.7 mL s cm^−2^, reducing 16.67%, 32.15% when compared with the fully stretched pristine fabric and the folded pristine fabric, respectively, indicating the coated fabric still has pretty air permeability. Figure 5e shows the maximum stretching load required for the proposed fabric when the length increases 2.5 cm after stretching, including pristine fabric, fabric coated 1 time, 2 times, and 3 times. The testing setup is shown in Fig. S14. The weight of 1 time, 2 times, and 3 times coated fabric under folded state is 1305.05, 1762.97, and 1854.14 g m^−2^. Given the dual-mode capability of the fabric to switch between 3 and 2D configurations, excessive coating can fill the fabric's pores and restrict yarn movement, resulting in higher stiffness. This increased stiffness makes it more challenging to stretch the fabric, requiring greater external force to facilitate the transition between states. Consequently, the pristine fabric requires the minimum load, while the one coated 3 times requires the maximum load. The stretching recovery rate was also tested to evaluate the tensile elastic recovery. The setup is shown in Fig. S15. The fabric will be stretched repeatedly through a linear motor over 1000 cycles, and the length change of the fabric will be measured to determine the overall strain it can withstand. The results are shown in Fig. 5f. Stretching recovery rate after 0 h represents the length change rate measured immediately after the stretching is completed and stretching recovery rate after 24 h represents the length change rate measured 24 h after the stretching is completed. Immediately after the stretching is completed, the stretching recovery rate of the pristine is 1.42%, which is quite smaller than those coated fabric, whose stretching recovery rate reduces with the coating time. A possible reason is heating during the coating process can alter the recovery rate of the fabric, but with an increase in coating times, the coating fixes the position of the yarns, improving the stretching recovery performance of the fabric. After 24 h, the pristine fabric has reverted to its original shape, while the recovery rate of the coated fabric has significantly decreased to less than 5%. And then, after 48 h, the stretching recovery rate is so close to 0 that it can be considered to be negligible.

The washing test is also conducted. Results shown in Fig. 5g indicate that the weight of the fabrics increases with the increase of the coating times. However, the weight variation is quite small, showing that weight loss during washing can be negligible. Figure 5h demonstrates the tensile test result of the fabric. It is obvious that when the extension is smaller than 60 mm, the load is quite small, near 0. The reason is that the proposed fabric has a folded structure. In the initial stage, the extension of the fabric can seem like an unfolding process. Thus, the load required for the unfolding process is small and then the mechanical stress for the stretching is small, proving that it is easy to stretch the fabric. The second stage of the tensile test is the yarn stretching of the fabric. The maximum load occurs when the fabric breaks. The maximum load of the coated fabric is 245.9 N, 2.73× larger than the pristine fabric, indicating that the coatings can enhance the tensile strength of the fabric rather than weaken it. The water absorption and evaporation rate of the proposed and commercial fabric were measured. The results were shown in Fig. 5i. Due to the application of Coolmax yarn, the water absorption rate of the 3D fabric can reach 387.89%, which is larger than the commercial fabric, 210.41%. The evaporation rate of the 3D fabric is 1.182 g h^−1^, larger than the commercial fabric which is 1.035 g. Figure 5j presents a radar chart for comparison among various textiles. Although there are existing works on textiles for personal thermal management, the textiles designed in this work can simultaneously address a broader application range, breathability and highly controllable, scalable manufacturing.

## Conclusion

In summary, an all-weather self-folding 3D knitted fabric with dual mode has been developed for personal warming and radiative cooling. The fabric is designed to self-fold, allowing it to transition between 3 and 2D states. This transformation enables thickness variations, resulting in adjustable thermal resistance. Part of the 3D fabric is coated with TiO_2_ and PDMS. TiO_2_ particles enhance the fabric's solar light reflectance, while PDMS provides higher emissivity in the 8–13 µm range, boosting heat dissipation through radiation. In warming mode, the entire fabric, including both the pristine and coated parts, retains a 3D structure that traps still air between layers. This increases thermal resistance, helping to maintain body temperature in cold environments. When solar light intensity increases, the fabric switches to cooling mode. The coated part can be fully stretched to cover the skin, reflecting solar irradiance. This transformation into a thin, unfolded layer allows more heat to escape, thereby reducing the skin surface temperature. Moreover, the fabric maintains excellent air and water permeability, ensuring high wear comfort. The folding and unfolding process requires only minimal strain, making the transition easy and efficient. Overall, this dual-mode fabric offers a practical solution for efficient warming and radiative cooling, adaptable to rapidly changing conditions.

## Supplementary Information

Below is the link to the electronic supplementary material.

Supplementary file1 (DOCX 3041 KB)
