# Supplementary material for: All-Weather 3D Self-Folding Fabric for Adaptive Personal Thermoregulation
Source: Nanomicro Lett. 2025 Jun 12;17:290. doi: 10.1007/s40820-025-01812-2 (PMC12158874; doi:10.1007/s40820-025-01812-2)
Supplement: Supplementary file 1 — Supplementary file1 (DOCX 3041 KB) [file 40820_2025_1812_MOESM1_ESM.docx]

Supporting Information for

**All-Weather 3D Self-Folding Fabric for Adaptive Personal Thermoregulation**

Xiaohui Zhang ^1,2,3^, Yuheng Gu ^1,2,3^, Xujiang Chao ^5^, Zhaokun Wang ^1,2,3^, Shitong Wu ^1,2,3^,

Jinhao Xu ^1,2,3^, Ziqi Li ^1,2,3^, Mengjiao Pan ^1,2,3^, Dahua Shou ^1,2,3,4^*

^1^ Future Intelligent Wear Centre, School of Fashion and Textiles, The Hong Kong Polytechnic University, Kowloon, Hong Kong, 999077, P. R. China

^2^ Research Centre of Textiles for Future Fashion, The Hong Kong Polytechnic University, Hung Hom, Kowloon, Hong Kong, 999077, P. R. China

^3^ Research Institute for Intelligent Wearable Systems, The Hong Kong Polytechnic University, Hung Hom, Kowloon, Hong Kong, 999077, P. R. China

^4^ PolyU-Xingguo Technology and Innovation Research Institute, The Hong Kong Polytechnic University, Hung Hom, Kowloon, Hong Kong, 999077, P. R. China

^5^ School of Mechanical Engineering, Northwestern Polytechnical University, Xi’an 710072, P. R. China

*Corresponding author. E-mail: [dahua.shou@polyu.edu.hk](mailto:dahua.shou@polyu.edu.hk) (Dahua Shou)

**S1 Numerical modelling analysis of warming performance**

The thermal insulation properties of materials are decided by thermal conductivity or the thermal resistance. Thermal conduction is the transfer of internal energy by movement of electrons or lattice vibration within a body and microscopic collisions of particles from a high-temperature area to a low-temperature region between two adjacent materials. Thermal conduction mechanisms, mainly including electronic transport and phonon transport, are dependent upon lattice structures and chemical components. To maintain the core temperature of the human body within a narrow range, proper clothing assists in both heat dissipation from the body skin to the surroundings and thermal insulation against extremely cold or hot conditions. Fourier’s Law states that the rate of heat transfer through a medium is proportional to the negative gradient in the temperature, viz.,

$Q=-\lambda A\cdot\frac{\partial T}{\partial x}$ (S-1)

where Q is the heat flux, λ is the thermal conductivity coefficient the system, and ∂T/∂x is the temperature gradient. A is the area. In a steady state, heat transfer is mainly characterized by effective thermal conductivity, which can be controllable by introducing highly conductive composite fillers or creating highly porous structures filled with thermally insulative phases such as encapsulated still air.

**S2 Numerical modelling analysis of radiative cooling performance**

This section provides in-depth analysis of models and calculations of PRC, which are the fundamental steppingstones for accurate assessments of the cooling magnitudes of various types of cooling systems, including radiative cooling systems. The net cooling power (P_net_) associated with radiative cooling, a primary focus of this section, is calculated based on several key factors, including the outgoing radiative power generated by the cooling structure (P_rad_), the amount of atmospheric radiation (P_atm_) that is incident upon the structure, the solar irradiance (P_sun_) that is incident upon the structure, and the nonradiative heat (P_nonradiative_) that is absorbed by the structure when considering the surrounding effects.


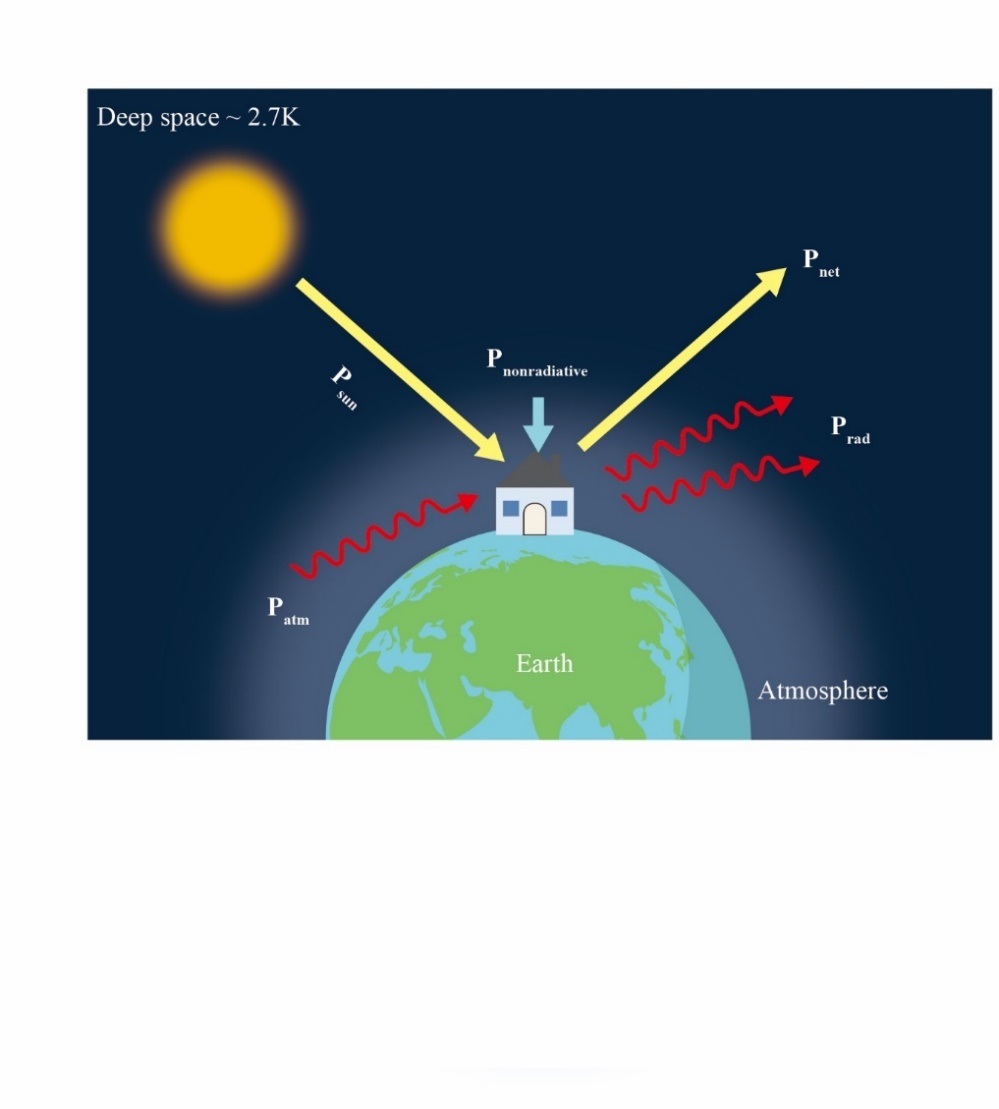


**Fig. S1** Schematic of energy flows into and through a radiative structure.

The heat balance of the radiative cooler can be described in Eq. (S-2) to Eq. (S-8) and the schematic is shown in Fig. S1.

$P_{net}=P_{rad}-P_{atm}-P_{sun}-P_{nonradiative}$ (S-2)

Where, $P_{rad}= 2\pi\int_{0}^{\frac{\pi}{2}} sin \theta cos \theta\int_{0}^{\infty} I_{B}(T_{S},\lambda)\varepsilon(\lambda, \theta)d\theta d\lambda$ (S-3)

$P_{atm}= 2\pi\int_{0}^{\frac{\pi}{2}} sin \theta cos \theta\int_{0}^{\infty} I_{B}(T_{amb},\lambda)\varepsilon(\lambda, \theta)\varepsilon_{atm}(\lambda, \theta)d\theta d\lambda$ (S-4)

$I_{B}\left( T,\lambda\right)=\frac{{2hc}^{2}}{\lambda^{5}(e^{\frac{hc}{\lambda kT}}-1)}$ (S-5)

$\varepsilon_{atm}\left( \lambda, \theta\right)=1-t{(\lambda)}^{\frac{1}{cos\theta}}$ (S-6)

$P_{sun}=2\pi\int_{0}^{\infty} I_{\mathrm{sun}}(\lambda)\varepsilon(\lambda, \theta_{sun})d\lambda$ (S-7)

$P_{nonradiative}=h_{c}(T_{amb}-T_{s})$ (S-8)

Where $T_{S}$is the surface temperature of the material. $T_{amb}$ is the ambient temperature, $\varepsilon_{atm}$ represents the spectral and angular atmospheric emittance. The spectral and angular emissivity of a radiative cooler, ε(λ, θ), is a key factor that determines the amount of heat dissipated from the device. Additionally, the atmospheric transmittance, t(λ), and the atmospheric emissivity, ε_atm_(λ, θ), are functions of wavelength and zenith (θ) and account for the absorption of radiation by the atmosphere. The combined convection and conduction heat transfer coefficient, hc, also influences the rate of heat loss from the cooler.

According to the energy balance theory above, the net cooling power is the comprehensive manifestation of the four factors. In order to achieve passive cooling property, the P_atm_, P_sun_ and P_nonradiative_ should be minimized. Specifically, the surface of the materials must be designed to have strong emission at the wavelength range between 8 and 13 μm, which corresponds to a region of the electromagnetic spectrum where the atmosphere is transparent. In addition, the coating must exhibit high reflectivity at visible and near-infrared (Vis-NIR) wavelengths, which are typically utilized for astronomical observations.

**S3 Design of the proposed garment**

Fig. S2a illustrates the garment in its warming mode, where the fabric remains in a folded state, increasing its thickness to enhance thermal insulation. In contrast, Fig. S2b shows the garment in cooling mode, with the fabric fully stretched to cover more skin surface. This stretching naturally increases the garment's length. To address this, the excess fabric, which is the pristine part, is strategically gathered at the shoulders, wrists, and sides of the body fixed by buttons. These areas typically experience higher perspiration. By incorporating Coolmax yarn, the garment not only absorbs sweat more effectively but also enhances evaporation, thereby keeping the wearer cooler. Fig. S2c-d shows the arm with sleeves in dual mode. The buttons are used to fix the gathered excess.


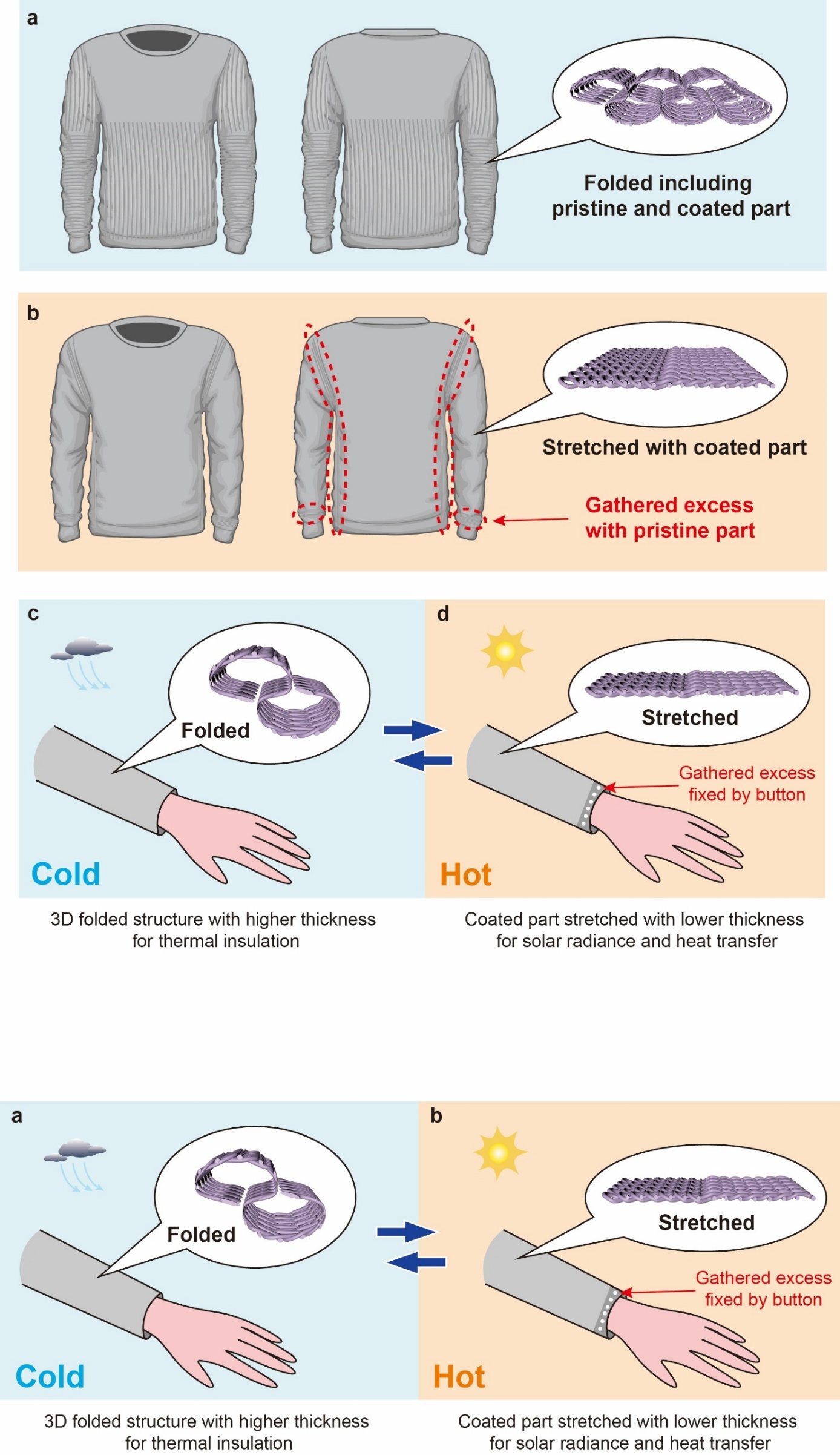


**Fig. S2** Garment fabricated by self-folding 3D knitted fabric. **(a)** Garment in warming mode. **(b)** Garment in cooling mode

**S4** **Preparation of the dual mode fabric**

The proposed dual mode fabric was fabricated by computational flat knitting machine with self-folded structure, whose folding mechanism can be explained in Fig. S3. Fig. S3a, b shows the single front and back loop, respectively. Fig. S3c, d displays the curling effect of the fabric made by all front and back stitches, respectively. Fig. S3e is the combination of front and back stitches which is called 7×7 garter as there are 7 courses of front and 7 courses of back stitches.

The knitting notation of the proposed fabric can be found in Fig. S3f. The front stitch is fabricated on the front needle bed and the back stitch is formed on the back needle bed. During the fabrication process, the yarn will knit on the front needle bed first and complete *K* courses. Then, the yarn will knit on the back needle bed and complete *M* courses. Finally, the loops on the back needle will be transferred to the front needle. One unit is completed with *K* (front stitches) + *M* (back stitches) courses. The value of *K* and *N* is generally the same to maintain the stable structure of the fabric.


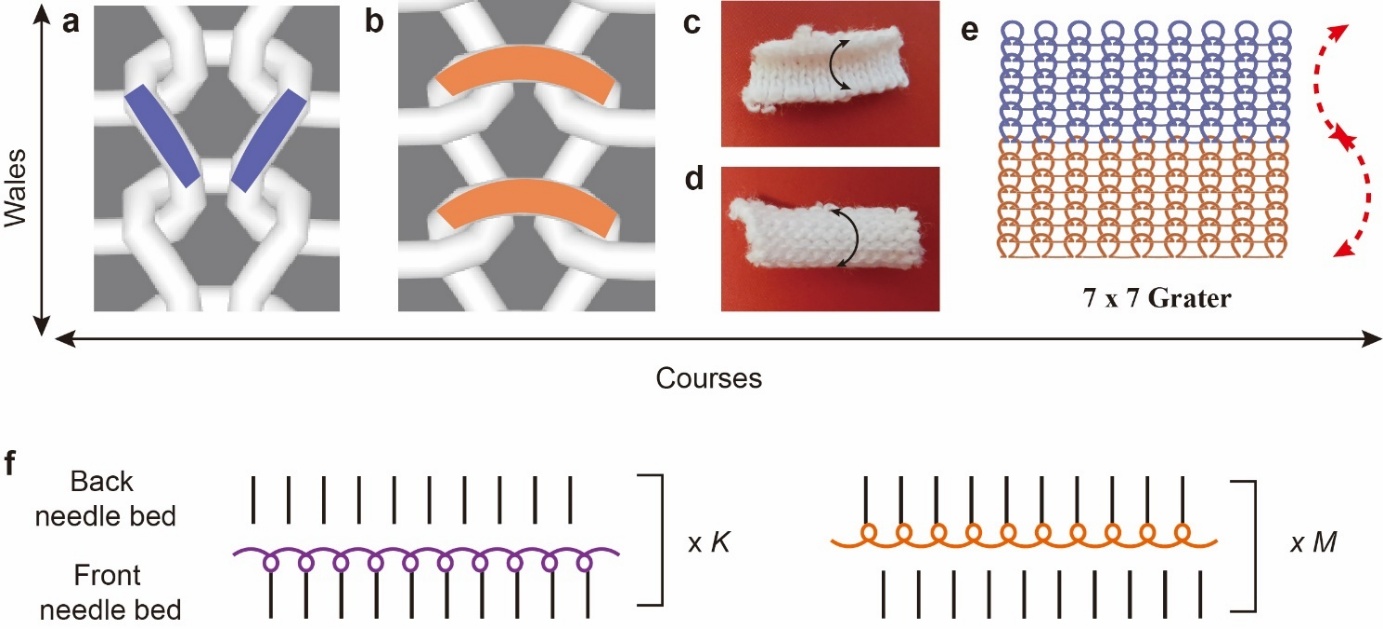


**Fig. S3** Knitting structure. **(a)** Single front loop. **(b)** Single back loop. **(c)** Curled single jersey knit with front stitches as the upper face. **(d)** Curled single jersey knit with back stitches as the upper face. **(e)** Knitting loops of 7x7 garter. **(f)** Knitting notation of the knitted fabric. The needles knit on the front and rear needle bar alternately to form front and back loops

The loop structure in one unit of the samples fabricated in this research is shown in Fig. S4a and the surface photography can be found in Fig. S4b. With the increase of the courses in the single unit, the length of the surface also rises.


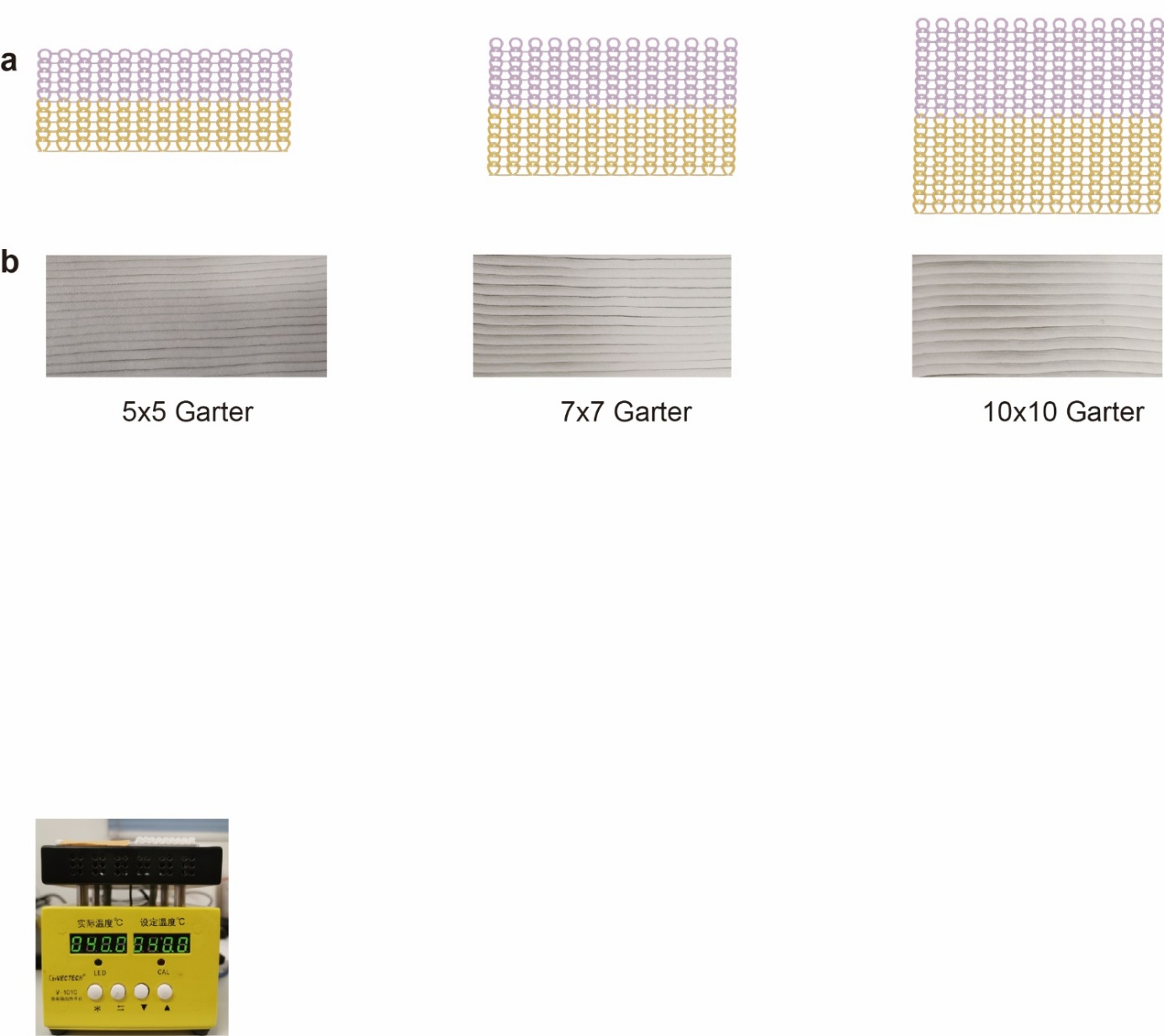


**Fig. S4** Schematic and the photography of proposed knitted fabric. **(a)** The loop structure of the knitted fabric in one single unit. From left to right, they are 5×5 garter, 7×7 garter and 10x10 garter. **(b)** The upper surface of the three samples. From left to right, they are 5×5 garter, 7×7 garter and 10×10 garter


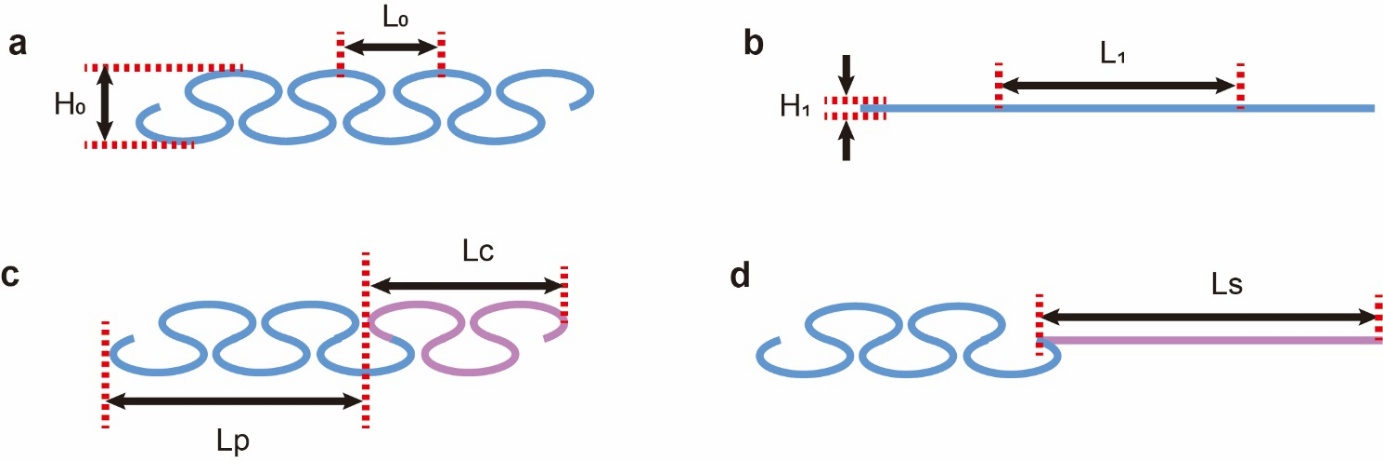


**Fig. S5** A simplified mathematical model illustrating the deformation of the dual mode fabric. **(a-b)** Length and height variations of fabric under folded and stretched state. L_0_ represents the initial length of the fabric under folded state, and L_1_ means the length of the fabric after stretched. H_0_ and H_1_ indicate the height of the fabric under folded and stretched state, respectively. **(c-d)** Length ratio of coated and pristine fabric under folded and stretched state. L_c_ indicates the length of coated part. L_p_ represents the pristine part. L_s_ represents the length of skin that requires to be covered

For 7×7 garter, if L_0_ is 10mm, L_1_ is 24 mm, which means that the fabric measures a compact 10 mm when folded and can stretch to a generous 24 mm. If the length of the skin requires to be covered is L_s_, the length of fabric in folded state (L_c_) and the stretched coated fabric (L_cs_) should be the same to ensure thermal performance.

$L_{cs}= \frac{12}{5}L_{c}$ (S-9)

$L_{s}=L_{p}+L_{c}=L_{cs}$ (S-10)

Then,

$L_{p}=\frac{7}{5}L_{c}$ (S-11)

Therefore, the ratio of the pristine and coated part of the dual mode fabric should be 7:5 (L_p_:L_c_).

**S5 Characterization of self-weight elongation**

Three samples with the same length (50 cm) and width (35 cm) were selected. Firstly, the fabrics were placed on a horizontal plate and any arbitrary length is selected and marked as E_0_, which serves as a reference point for subsequent measurements. Then, the top edges of the fabric were fixed, and the fabric hangs freely with no external forces acting upon it. The experimental setup can be found in Fig. S6. The selected length would increase due to the weight, which is measured as E_1_. The self-weight elongation can be calculated by the formula below.

$self-weight elongation \left( \% \right)= \frac{E_{1}-E_{0}}{E_{0}}$ (S-12)


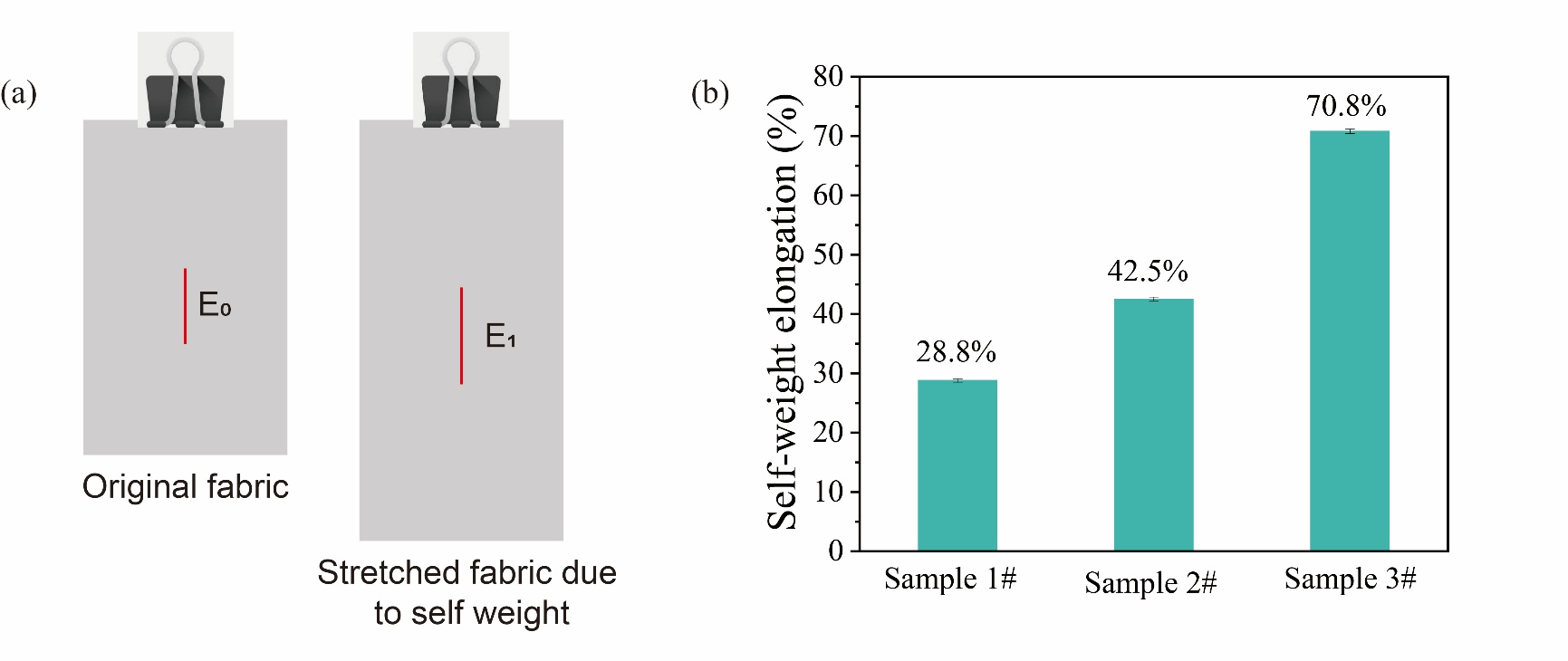


**Fig. S6** Elongation of the fabric due to self-weight. A clip is used to fix the top end of the fabric. The length variations of these three samples are illustrated

**S6 Evaluation of the thermal and moisture resistance**

The thermal and moisture resistance were both measured by the sweating guarded hotplate according to the modified ISO 11092:1993 Textiles - Physiological effects - Measurement of thermal and water-vapor resistance under steady-state conditions (sweating guarded-hotplate test). The size of the hotplate is 50 cm×50 cm. The setting up of the samples can be seen in Fig. S7.


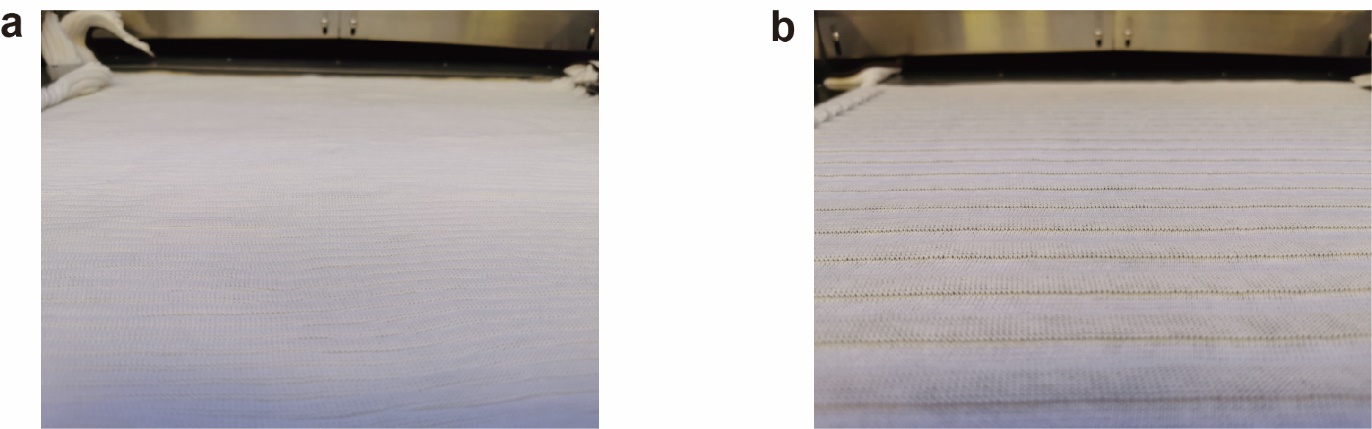


**Fig. S7** Setup of the thermal and moisture resistance of different samples, including 5×5, 7×7 and 10×10 garter. **(a)** Folded fabric during the testing. **(b)** Fully stretched fabric during the testing

**S7 Thermal images of the fabrics**

The thermal images in Fig. S8, along with the collected thermal resistance data, indicate that the thermal performance of the fabric in both its stretched and relaxed states is comparable to that of a lightweight long-sleeve shirt and a thick jacket.


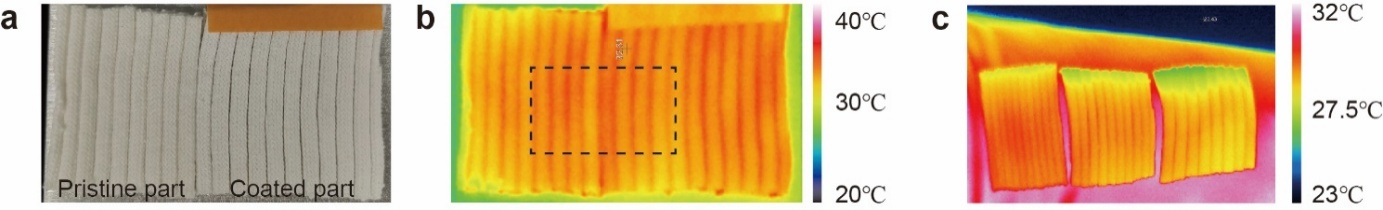


**Fig. S8** **(a)** Upper surface photograph of the proposed fabric in folded state on the hotplate. The left side is the pure part, and the right part is the coated part. **(b)** Thermal image of the fabric on hotplate. The middle part selected has the same temperature. **(c)** Thermal images of the three samples placed on human arms. From left to right, they are 5x5 garter, 7x7 garter and 10x10 garter

**S8** **SEM images of fabric with different particle weight ratio and coating times**

In order to observe the particles on the surface of the fabric, SEM images of fabric with different particle weight ratio and coating times were collected, shown in Fig. S9.


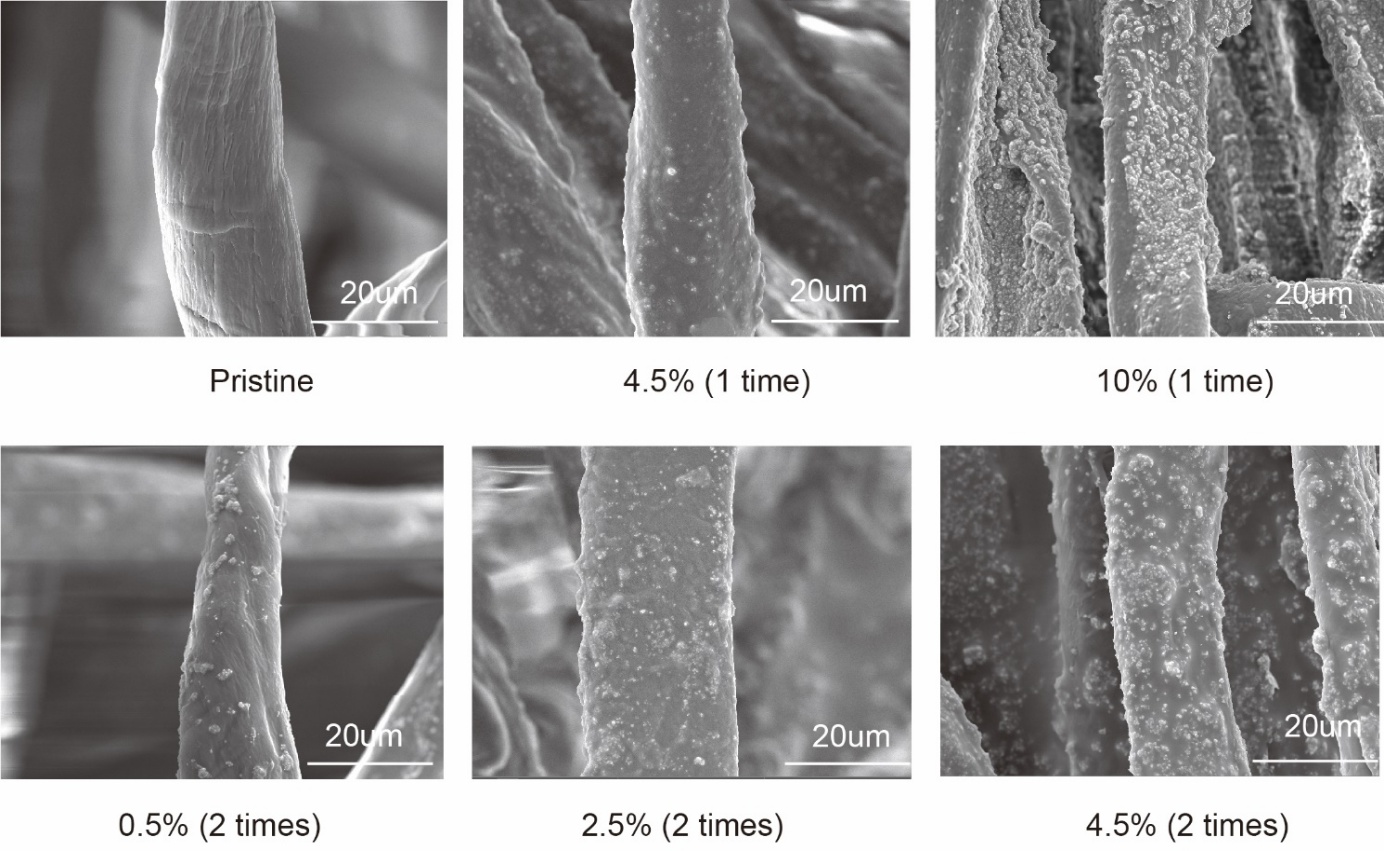


**Fig. S9** SEM images of fabric with different weight ratio and coating times. The particle number increases with the increase of the weight ratio and the coating times

**S9 Reflectance of the proposed fabric with different particle size and coating times**

**Table S1** The reflectance of the 7x7 garter with different particle size and coating times

| Sample | 2 coating times | 3 coating times |
| --- | --- | --- |
| 500 nm, 4.5% | 89.5% | 89% |
| 200 mm, 4.5% | 87% | 88% |
| 1µm, 4.5% | 87% | 88% |

**S10 FDTD simulation to investigate the effect of TiO_2_/PDMS coating on reflectance**

A finite-difference time-domain (FDTD) simulation is applied to study the effects of particle size on the optical properties. Lumerical FDTD Solutions software was used to perform FDTD simulation. A two-dimensional model was set up to simplify the analysis and reduce the simulation time. The range of 0.25–2.5 μm (Solar range) was selected to study the NIR reflectivity enhancement of the coating caused by the scattering effect. The size of the TiO_2_ particles ranges from 0.1-1.1 μm. The PDMS was selected as the matrix.

**S11 Radiative cooling performance of the fabric under solar simulator**


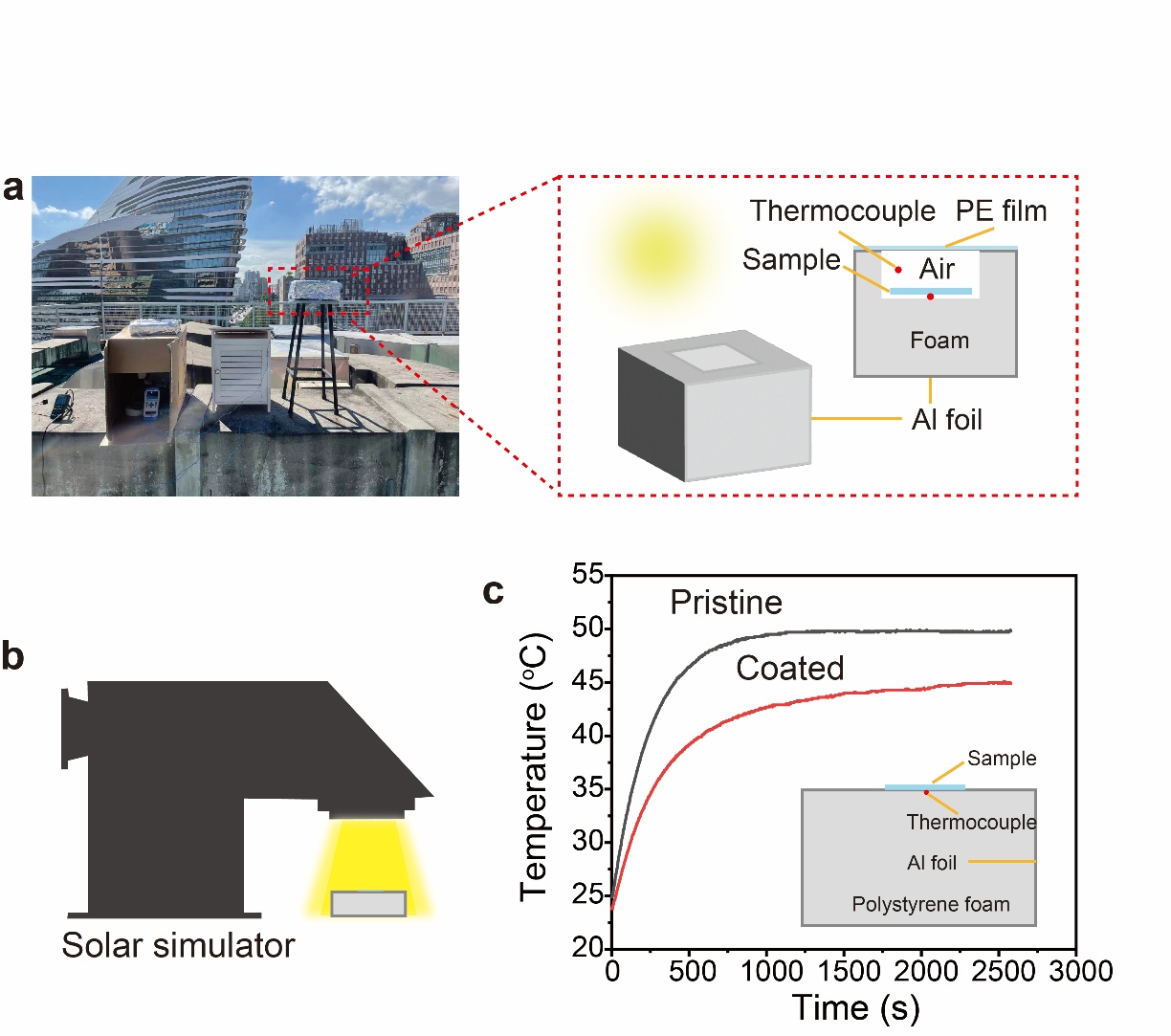


**Fig. S10** Outdoor and indoor thermal measurement setup and the results. **(a)** Diagram of the outdoor thermal measurement setup. **(b-c)** Indoor thermal measurement system used to evaluate the radiative cooling properties and temperature collected during the tests.

**S12 Dual mode textile-based sleeve**

As shown in Fig. S11, the two ends of the sleeve are fixed with elastic binding to ensure stability. Besides, another elastic binding is placed between the edges of coated and pristine part. In warming mode (Fig. S11a), the sleeve is folded to prevent heat loss from body. In cooling mode (Fig. S11b), the coated part is stretched by adjusting this binding placed between the coated and pristine parts. The pristine parts shift to the wrist or the side of the body for fast sweat absorption and evaporation.


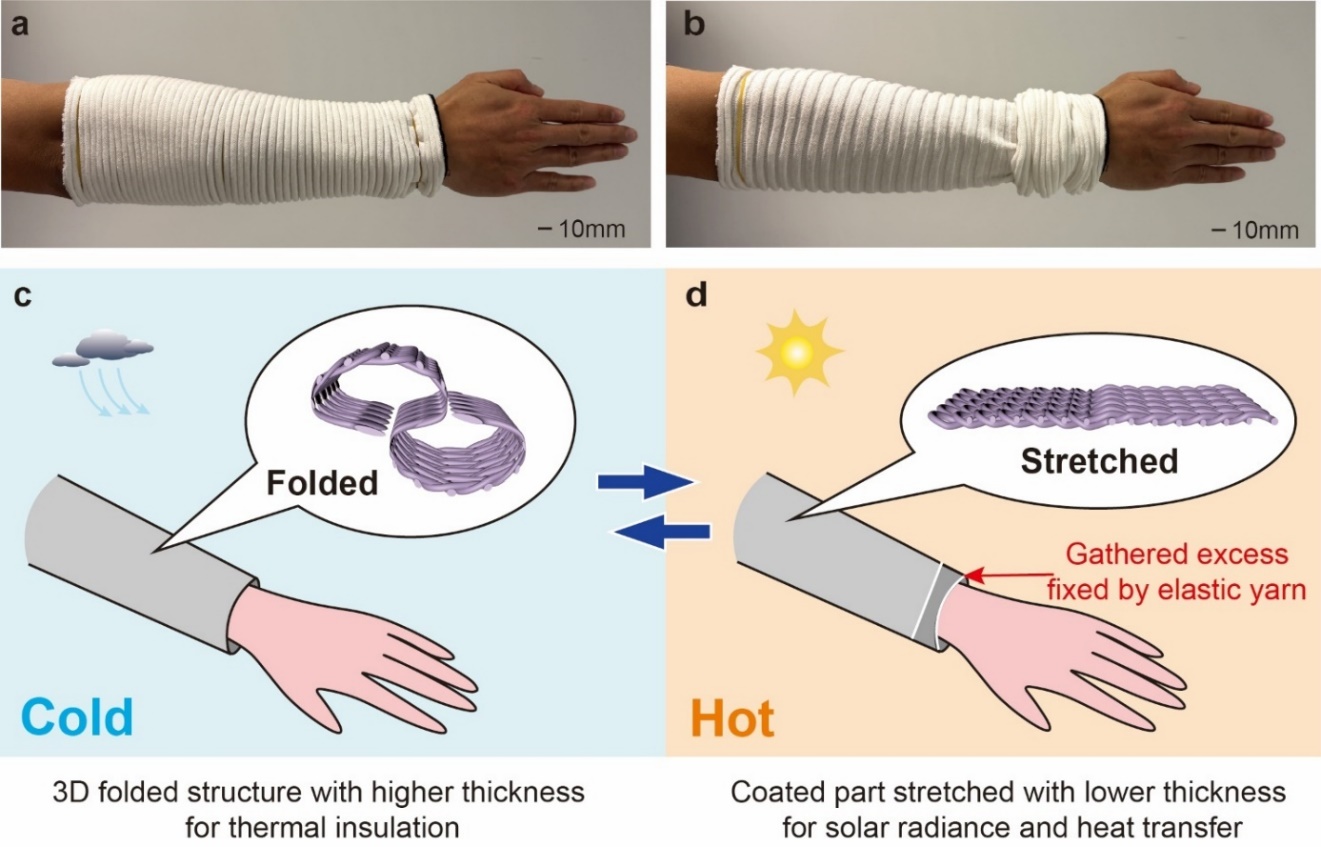


**Fig. S11** Forearms covered with dual mode textile-based sleeve. Real images of the arm covered with sleeve in warming mode **(a)** and cooling mode **(b)**. Schematic of the arm with sleeves in warming mode **(c)** and cooling mode **(d)**

**S12 Water vapor loss testing**


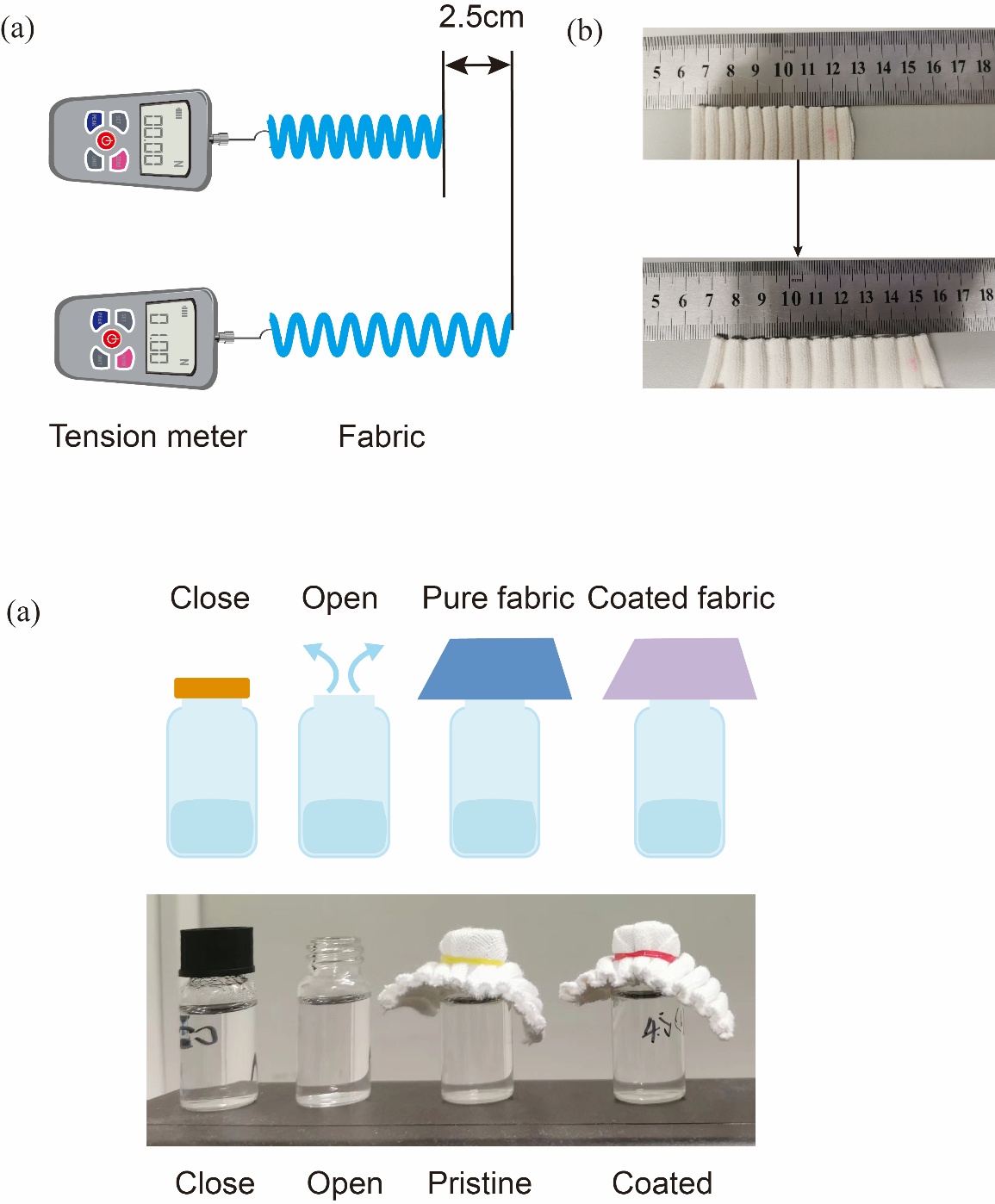


**Fig. S12** Setup to test the water vapor loss, including closed bottle, open bottle, bottle covered by pristine and coated fabric (4.5%, 2 times)

**S13 Flexibility and stretchability of the coated fabric**

The flexibility of the fabric samples was assessed through a series of bending tests, with the results detailed in Fig. S13. The findings indicate that, as coating time increases, the bending angle decreases. The findings suggest that additional coating actually reduces flexibility, resulting in increased stiffness of the fabric, which makes it less able to bend easily. This behavior likely occurs because the coatings alter the fabric's surface characteristics, enhancing its resistance to deformation and potentially affecting its structural integrity.


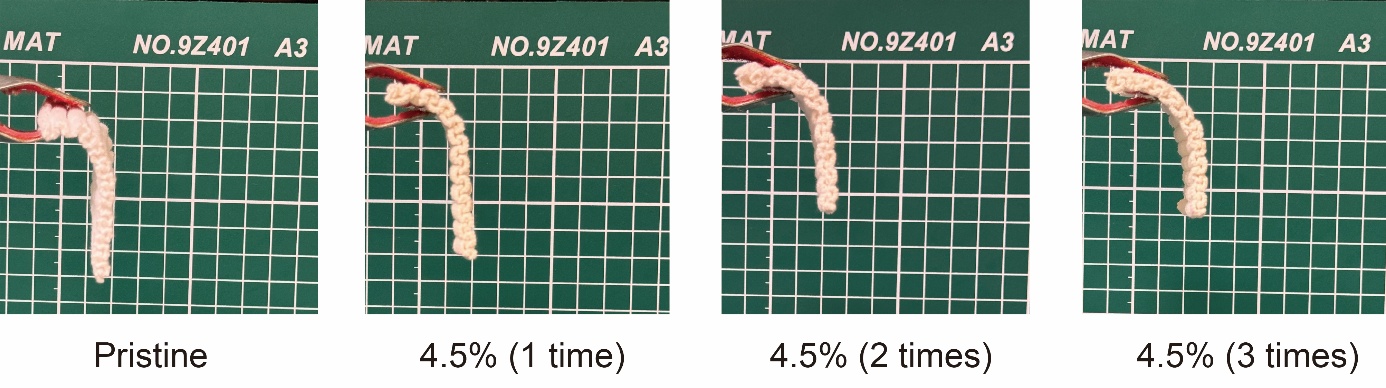


**Fig. S13** Bending test results of fabrics with various coating times

The stretchability of the coated fabrics was examined. As shown in Fig. S14, a tension meter was utilized to evaluate the extent to which the coated fabrics could be stretched. Specifically, the fabric gradually extended until it had expanded by approximately 2.5 cm. At this point, the tension exerted on the fabric was precisely measured. The results can be found in Fig. S14.


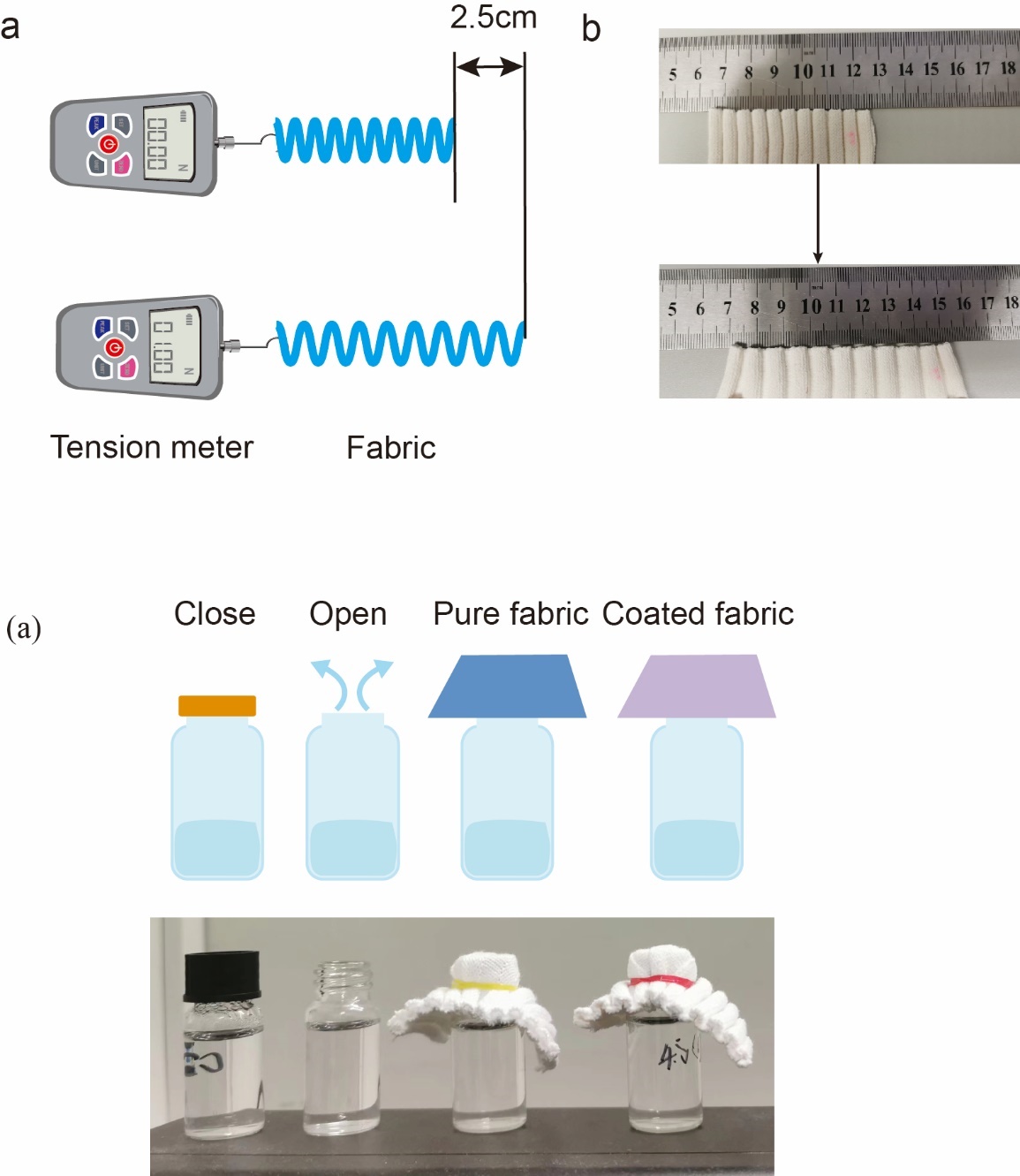


**Fig. S14** Maximum load required for the proposed fabric when the length increases 2.5 cm after stretching, including pure fabric, fabric coated 1 time, 2 times and 3 times

**S14 Stretching recovery testing**

The stretching recovery performance of the proposed dual mode fabric was evaluated by stretching the fabric 1000 cycles to observe the length variation. The setup is shown in Fig. S15. One cycle includes a fully stretching process and recovery progress. The size of the fabric is 6.1 cm × 5.5 cm.


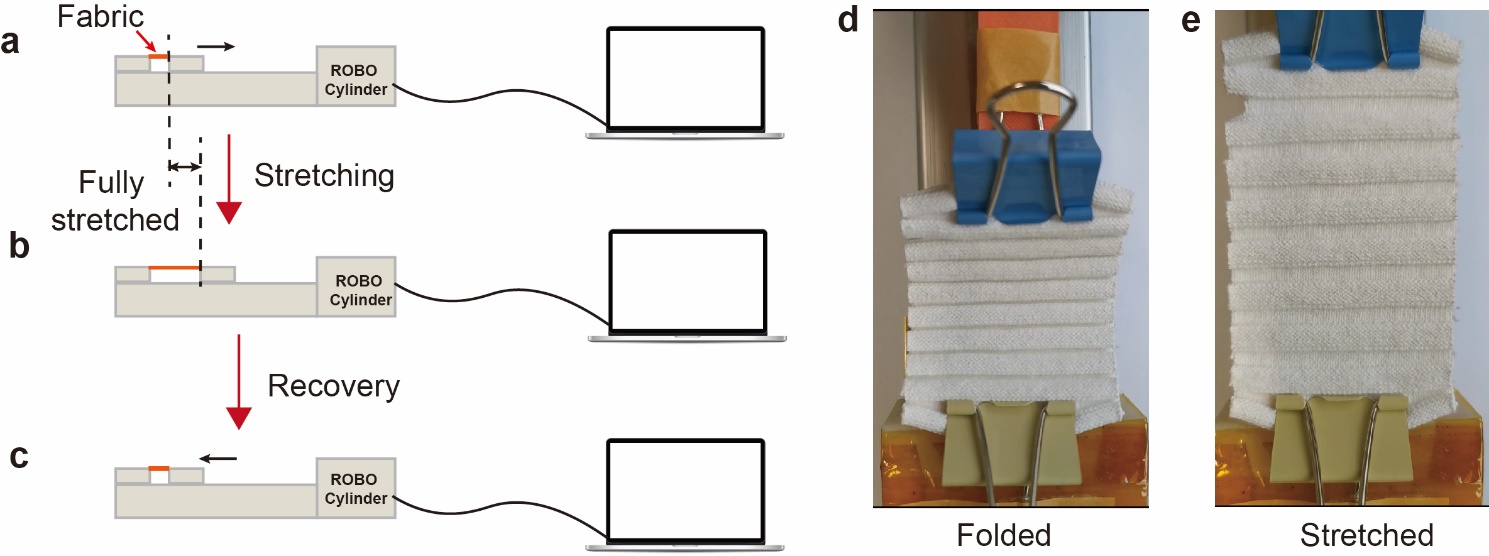


**Fig. S15** Stretching recovery testing of the proposed fabric by a cylinder within 1000 cycles. **(a-c)** 1 cycle of stretching recovery process
